# Acquisition of a new language: an *enriched* case study documents language growth without external input in a young Korean child’s acquisition of English

**DOI:** 10.3389/fnhum.2024.1456054

**Published:** 2024-11-27

**Authors:** Barbara Lust, Suzanne Flynn, Ahyoung Alicia Kim

**Affiliations:** ^1^Psychology, Cornell University, Ithaca, NY, United States; ^2^Linguistics and Philosophy, Massachusetts Institute of Technology, Cambridge, MA, United States; ^3^WIDA, University of Wisconsin-Madison, Madison, WI, United States

**Keywords:** Korean, English, bilingualism, second language acquisition, maturation, language input

## Abstract

This paper explores a case of suspension of data input during the acquisition of a second language by a young Korean child acquiring English in an English-only nursery school in the United States. Data suspension occurred naturally when the child returned to Korea for a summer where only Korean was spoken. Systematic investigations using an enriched case study methodology which assessed the nature of the child’s English target language acquisition both before and after the Korean Summer revealed significant advances in his English after the Korean Summer despite the absence of English input during this time. Several hypotheses regarding the nature and explanation of this advance are tested. It is argued that significant internal linguistic integration leading to systematization of linguistic knowledge occurred in the absence of synchronous language data input, demonstrating the significance of internal computational processes over and above language data input in the language acquisition process. Results have implications for understanding the fundamental nature of language acquisition.

## 1 Introduction

Perhaps the most fundamental issue in cognitive science concerns the degree to which, and the manner in which, language experience is critical to the acquisition of a target language. Specifically, what is the relation of input data to internal language creation by the human mind? This issue has wide implications for both the cognitive science of language acquisition [e.g., in “Poverty of the Stimulus” argumentation (Berwick et al., 2011; Pearl, 2022; Gleitman et al., 2019 for a recent review)] and the science of education. (See also Crain, 1991 and commentary for debate in the field of language acquisition.) However, investigation of this issue is fundamentally challenging, as in the canonical case, for example, of a child acquiring a first language (or languages), language creation (internal computation), and tangible input from the environment are both necessarily continuous, confounded and essentially immeasurable. Investigation of this issue is thus generally and necessarily limited to studies of pathological deprivation (e.g., Curtiss, 1977), or sign language exposure in deaf populations (e.g., Mayberry et al., 2002; Goldin-Meadow, 2003; Landau and Gleiman, 1985). Kegl (2002, 2021a,b) provides a summary of these issues and a study of the creation of language without initial language input as in Nicaraguan Sign Language.

In this paper, we exploit a recently developed methodology, an enriched case study, to investigate a case of the acquisition of spoken language bilingualism in a natural environment. Through this methodology, we examine the effects of suspension of language input during a young child’s acquisition of a new target language. Natural events during this case study provided us with a form of a *natural experiment*. By natural experiment, we refer to a case where the environment, not a researcher’s controlled planned experimental design, has naturally varied selected factors, in this case, data suspension in the form of interrupted language data input.

### 1.1 A natural experiment: the Korean summer

We report selected results from multiple assessments of a bright, active, friendly young Korean-born male, MJ, who was studied longitudinally in an English-only nursery school (Cornell Early Childhood Center; hereafter “ECC”) in the United States. MJ entered the ECC program (8 h/day) at age 2.9.0 (years, months, and days) and left at age 4.6.18. From birth, MJ’s parents continuously followed the rule, *of using only Korean at home*. In studying MJ, we will refer to English as the new target language (TL) or second language (L2) and Korean as the first language (L1).

Critically, during MJ’s enrollment at the English-only nursery school and during a period of important English language acquisition, MJ returned to Korea for the summer, a period of approximately 3 to 4 months. We refer to this event as the *Korean Summer.* During the Korean Summer, MJ was between 3.6 and 3.10 years of age.^1^ During this time, as attested by his mother, all English input for MJ was completely suspended. MJ was exposed only to Korean in Korea.

**Figure 1:**
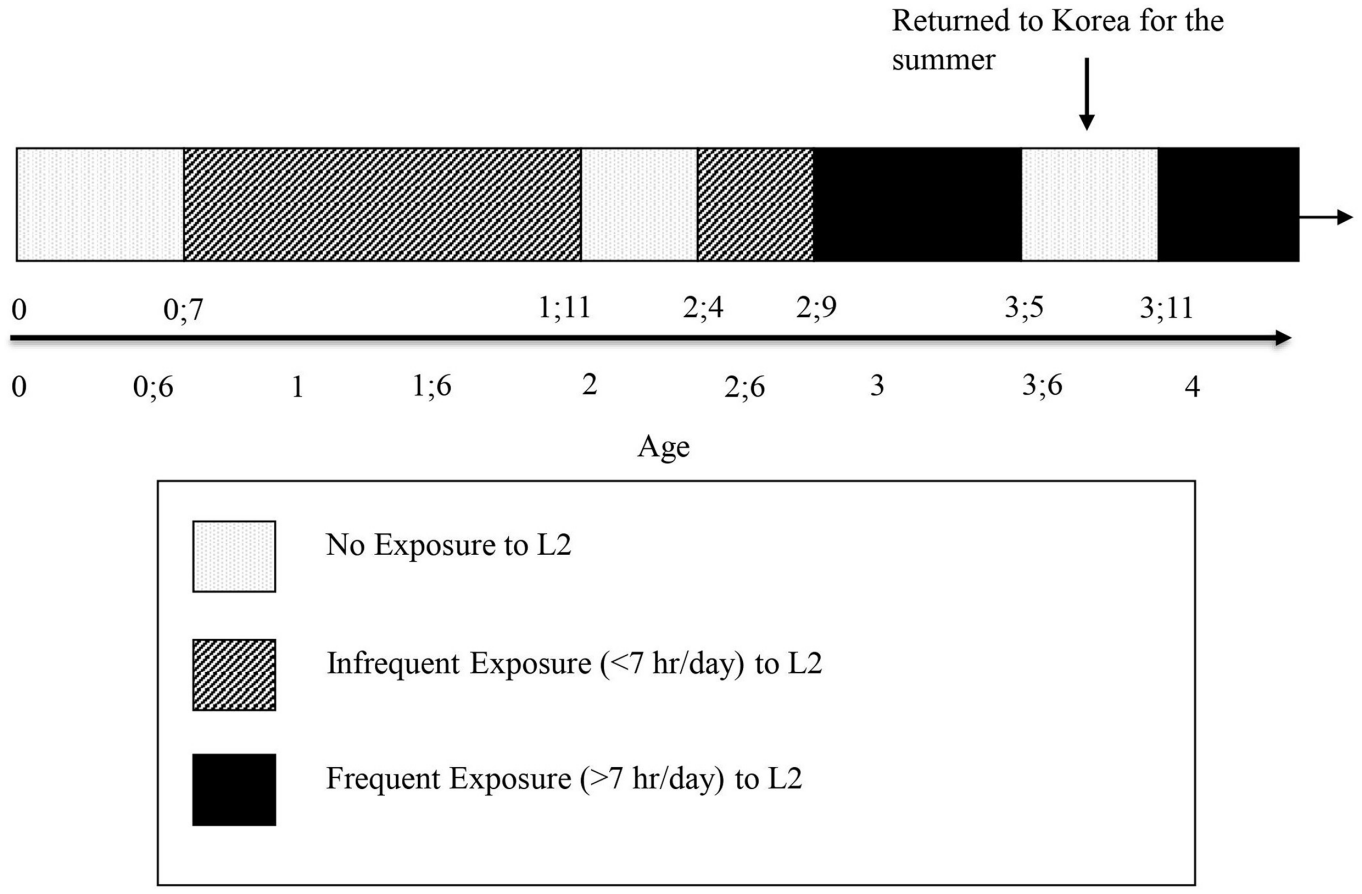
Exposure to L2 English by a young Korean child.

Figure 1 summarizes the history of language exposure for MJ, focusing on the periods of his exposure to English until he was 4.6.18, designating the period of the Korean Summer, when the use of and exposure to English was suspended.^2^

MJ’s Korean Summer provides us with a natural experiment; that is, this experiment occurs in a natural situation wherein all target language (English) data are eliminated in the midst of a child’s learning of that target language, even while the child’s physical and cognitive development proceeds normally.

Just before his summer departure, and approximately 5 months after entry to the English-only ECC and 5 months of English language nursery school immersion, Korean was used most of the time with his parents, and 70% with his Korean friends. At this time, MJ’s Korean was still “much better than his English” (Multilingualism Questionnaire results).

### 1.2 Research question

Based on this natural suspension of English language input for MJ during the Korean Summer, our leading research question thus became:

- Given the sustained experience without language input, i.e., the suspension of English language input over the Korean Summer, would this child’s English language acquisition show regression or attrition when the child returned to the English environment in the fall following his return from Korea?

In relation to this leading question, several hypotheses are possible. They, in turn, lead to several distinct predictions.

- Hypothesis 1: If the relation between language data input and language acquisition is direct, then, without continuous language reinforcement through continuous data exposure, MJ’s English language will show regression. As with many cognitive abilities, the absence of repeated experience may result in loss, e.g., language attrition.
- Hypothesis 2: If the relation between language data input and language acquisition is direct, but continual reinforcement is not necessary for that acquisition, then MJ’s stage of English language development will stabilize as it was before his Korean Summer.
- Hypothesis 3: If language acquisition significantly depends on internal computation above and beyond language input data, then, based on language acquisition to date, MJ may advance in language even without consistent data input. If this is the case, that is, if the relation between language data input and language acquisition is indirect, then MJ’s English language acquisition will continue to improve over the Korean Summer.

To address the fundamental research question stated above and the consequent hypotheses, we evaluated available data before and after MJ’s return to Korea, based on our *enriched case study* methodology, sampling from a critical set of assessments, summarized in Table 1.

**Table 1:** Assessments used in enriched case study.

Category	Type		Description
Parent/teacher reports	Multilingualism Questionnaire (MQ) (https://testvcla.cac.cornell.edu/the-vll/the-multilingualism-questionnaire/)		Parent/caretaker survey to gather metadata of child’s language **background** including selected socio-demographics
	Pragmatics [Pragmatic Profile (PP)] (Dewart and Summers, 1988)		Parent and /or teacher observations, designed to assess child’s **pragmatic** development
Child assessments	Naturalistic	General Observation (GO)	Naturalistic observations of child’s **pragmatic** and **grammatical** development
		Natural Speech (NS) (Blume and Lust, 2017)	From child’s natural speech, we focused on measuring **mean length of utterance** (MLU) and percentage of well-formed **grammatical** sentences
	Standardized	Lexico-Semantics [Peabody Picture Vocabulary Test (PPVT)] (Dunn and Dunn, 1997)	Standardized assessment of **vocabulary**
	Experimental	Syntax [Elicited Imitation (EI)] (Dye and Foley, 2020)	Experimental tests of **grammar/syntax** with a focus on complex sentence formation (coordination, relativization and adverbial subordinate clause formation)

In particular, Korean follows a subject–object–verb (SOV) sentence order, contrasting with English subject–verb–object (SVO) order and it differs from English in direction of embedding and adjunction in relativization and subordinate clauses (recursion direction). Functional categories such as complementizers appear clause finally. Korean and English differ not only in sentence structure but also in the distribution of function words and inflections. In Korean, verbs need not agree with the subject. In addition, overt articles such as “a, an” do not exist in Korean; therefore, nouns do not have to be introduced using articles. Rather, inflectional morphology is carried out by particles attached to the word stem. These particles can be used on nouns to showcase or theta role-marking and on verbs to indicate phenomena such as tense, mood, and honorifics. Thus, MJ’s target language acquisition reflects a complex new learning challenge involving multiple dimensions of language knowledge, e.g., syntax, order, morphology, nominal and verbal inflection, case and tense marking systems, and phonology.^3^

## 2 Method

For this study, we applied an *enriched case stud*y methodology wherein repeated naturalistic longitudinal observations of a single child are enriched by related periodic standardized and experimental tests.^4^ Our enriched case study method engages the strengths of naturalistic observations (by caretaker or researcher) while overcoming some of their limitations. The enriched case study methodology systematically integrates methods allowing (i) focus on particular research questions; (ii) integration with experimental designs testing hypotheses about specific forms of language knowledge; (iii) calibration with populations allowing a form of *normalization* beyond the case through standardized testing and through testing of comparable control individuals where available; (iv) and standardization of data and metadata collection, strengthening comparability across cases and populations; (v) considered together, the method provides converging evidence between naturalistic observations and experimentally derived results^5^ (see, for example, Lust et al., 2014).

### 2.1 Components of the enriched case study approach

Components of the enriched case study methodology are listed in Table 1 and briefly introduced in our Appendix. Results are entered into a structured standardized database to support archiving, analyses, calibrated reanalyses, and dissemination (Pareja-Lora et al., 2019).^6^

The enriched case study method reflects an idealized infrastructure for the study of language change over time within a single child.^7^ Its application (e.g., which assessments are given when and how often longitudinally) ultimately depends on the particularities of the individual, the case context, and the capacity and focus of the research team at any point in time. (See “Limitations” below.) This method provided critical evidence, which allowed us to evaluate MJ’s Korean Summer as a natural experiment, involving input data suspension during language development.

### 2.2 Procedures for assessing MJ

Applying our enriched case study methodology, MJ was assessed for a total of 49 sessions during his time in the ECC; beginning at age 3.0.23 and terminating at age 4.6.18; each session included one or more assessments. These assessments were conducted over approximately a year and a half.

**Figure 2:**
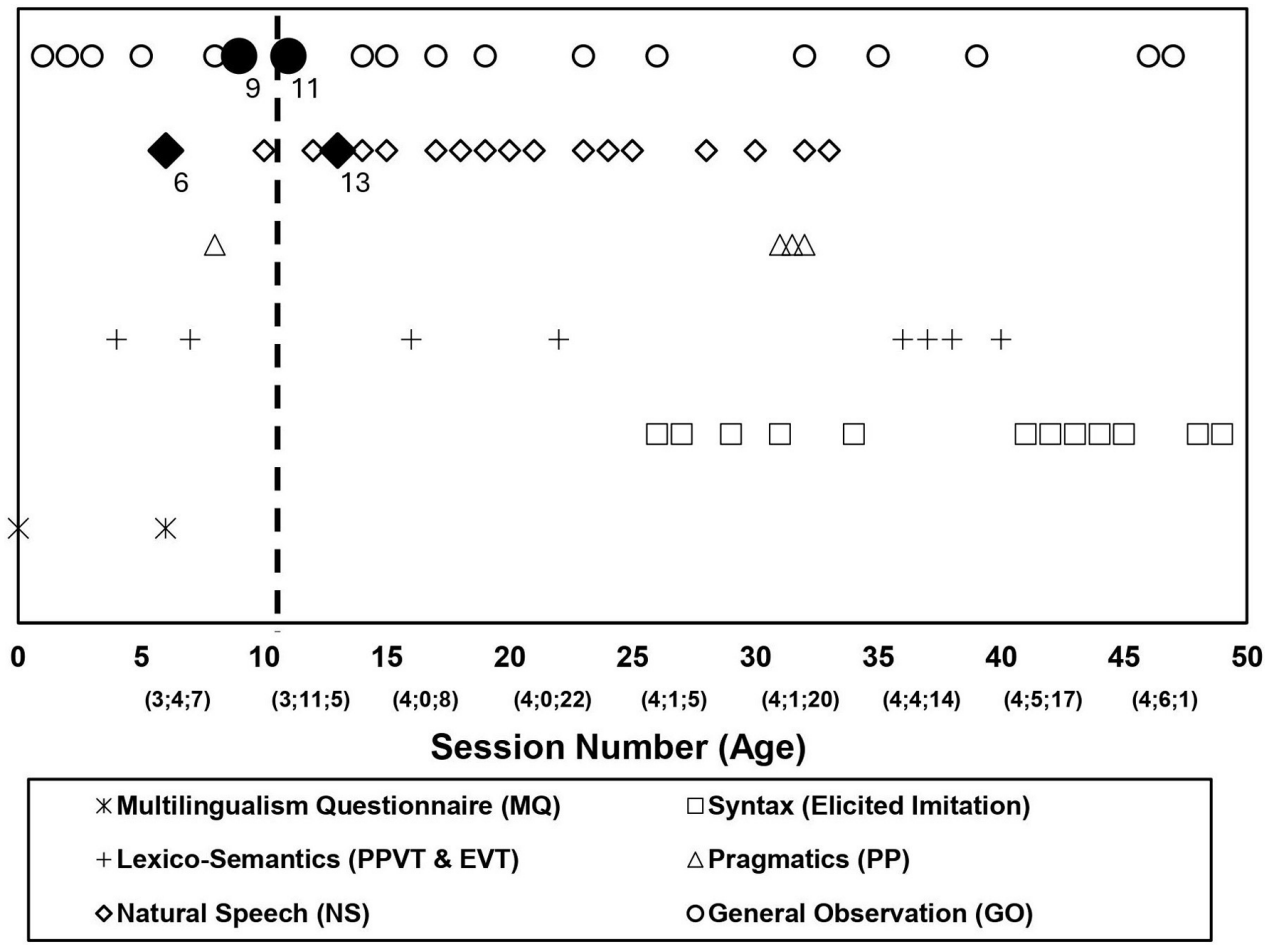
Assessment sessions over age.

Figure 2 summarizes the various assessments that were conducted over the 49 sessions in the period MJ was studied, identifying their session number from 1 to 49. A team of bilingual Korean–English speakers and English monolingual speakers were assigned to work with MJ as researchers during this period. Individual assessments were audio- and/or video-recorded, digitized, transcribed, and coded. Transcripts and coding were reliability-checked by multiple research team members.

In this paper, focusing on our hypotheses, we report selected results from across the 49 sessions and their multiple assessments, which bear directly on our research question, i.e., MJ’s language (English) development prior to and post the suspension of English language input during the Korean Summer. For this report, prior to MJ’s Korean Summer departure, we included evaluations of the general observations (GO) and natural speech data from MJ at assessment Sessions 6 (age 3.4.14) and 9 (age 3.5.28)—our last recordings *before* his Korean Summer—and compare these to those post-Korean Summer at Sessions 11 (age 3.11.19), 13 (age 4.0.1), and 17 (age 4.1.0), which closely follow MJ after his return in the fall.^8^ Overall, these sessions involved free play in the ECC classroom and one-on-one interactions with a researcher and/or teacher.

We supplement these results with selected data conducted over the full year and a half of cumulative study, which provide critical supplementary evidence on the general trajectory of MJ’s English language acquisition. These consisted of the Multilingualism Questionnaire (MQ), a Vocabulary Assessment (PPVT; Dunn and Dunn, 1997), and a Pragmatic Assessment (PP; Dewart and Summers, 1988). We include a report of the results from one elicitation imitation (EI) task on coordinate sentence structure, collected approximately 2 months post-Korean Summer. In this paper, given our leading research question, we focus on findings from assessments of MJ’s target language (English), although multiple assessments of MJ’s Korean language exist and are available for future study.^9^^,^^10^

The Multilingual Questionnaire^11^ completed by MJ’s mother when he began attending the ECC (repeated three times over the longitudinal course of the study) provided comprehensive background information about MJ and his language use and exposure. The MQ, administered when he began attending the ECC, confirmed that his Korean was developing well and was “fluent.” He is reported to have begun to “speak fluently” in Korean when he was 24 months old. At the time of his entry to the ECC, MJ’s English was “very limited,” while his Korean was “well developed.” MJ had difficulty comprehending TV programs broadcast in English. Overall, MJ was then estimated to produce Korean vs. English approximately 85% of the time.

## 3 Results

Our combined assessments, surprisingly, indicated that MJ’s target language acquisition (English) did not diminish over the period without English input, but rather, it significantly *improved*. MJ’s English language acquisition continuously developed both before and after the Korean Summer.

### 3.1 Vocabulary

**Figure 3:**
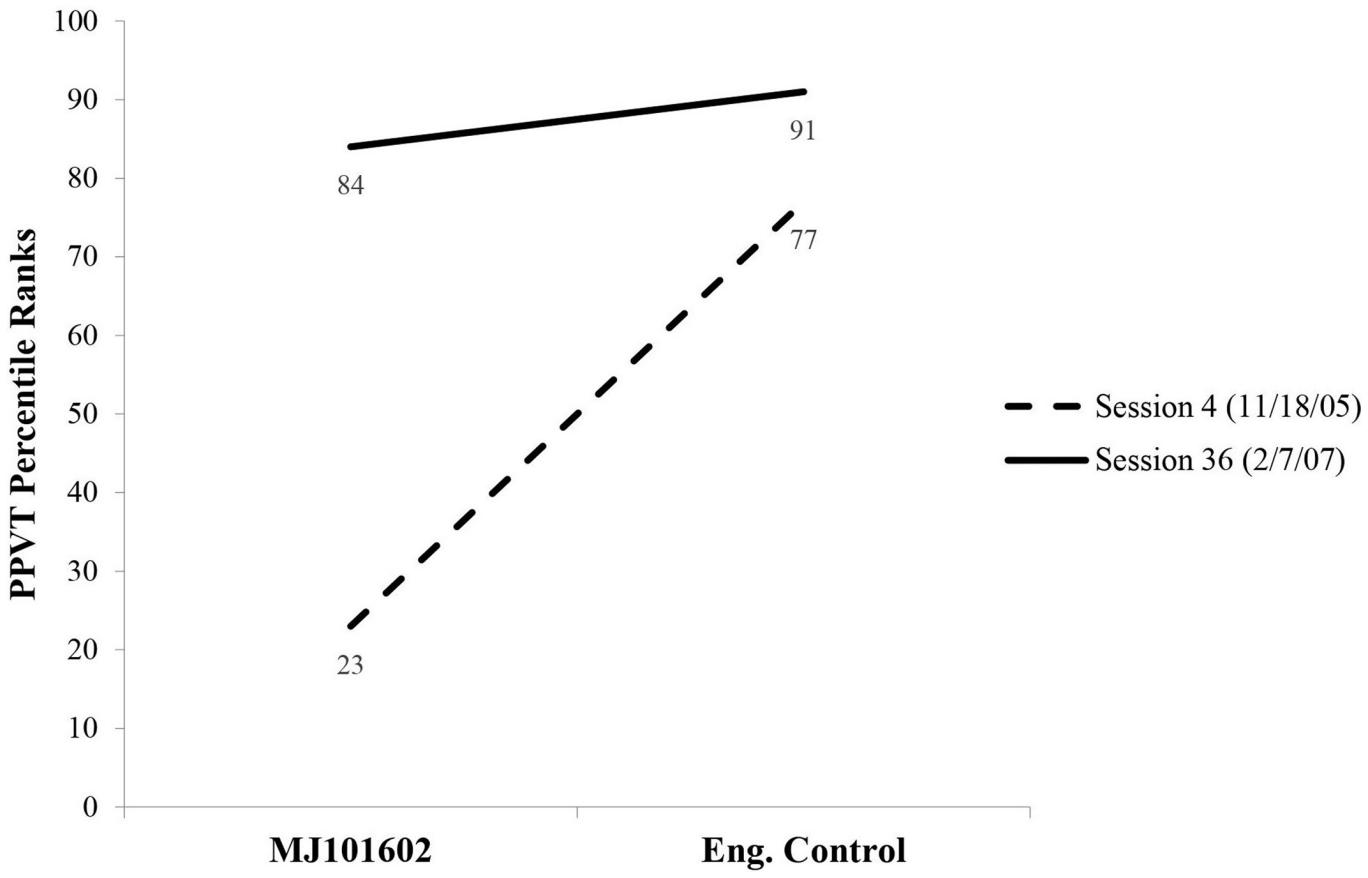
Performance of MJ on Peabody Picture Vocabulary Test at two points compared to monolingual English controls (Dunn and Dunn, 1997).

Results of the PPVT assessments provide normative evidence concerning MJ’s developing vocabulary comprehension during the time preceding the Korean Summer and then again after his return to the United States. Figure 3 compares MJ’s performance on the English PPVT administered soon after he enters the early childhood center (ECC) (Session 4 at age 3.1.2) to monolingual controls and to his performance when he was 4.4.19 years old (Session 36). As the figure indicates, on the first assessment, he ranks only at the 23^rd^ percentile in English relative to a standardized population. By the time he finally leaves the ECC (approximately age 4.6), he is virtually identical to the standardized monolingual English control population in terms of percentile rank, with no sign of deterioration.

### 3.2 Pragmatics: use of language

The *Pragmatic Profile* (PP) assessment provides parent and/or teacher observations on forms of MJ’s use of language: communicative functions (e.g., communicative intentions and responses) and interactions. It also provides important descriptive data from the point of view of a caretaker. Table 2 summarizes the PP data for MJ. As indicated, MJ increases dramatically in communicative language use over the time period studied, with a marked change in language use between the profile results as indicated before and after the Korean Summer. The PP data post-Korean Summer suggest a marked increase in MJ’s use of English in all communicative categories.

**Table 2:** Communicative functions and interaction: MJ before and after Korean summer (pragmatic profile results by teacher, Dewart and Summers, 1988)^a^.

	Session number (age; observer, situation)	
	Before Korean summer	After Korean summer
	Session 8 (age 3.4; teacher, classroom)	Session 32 (age 4.3; teacher, classroom)
Communicative functions^b^	Often only one or two words are in his short English utterances. He combines pointing to and naming persons/objects to get attention to himself (“look”) and needs and desires (“more” or “again,” “what,” “what’s that”) He greets peers and teachers (“goodbye”), names objects and is attempting to learn English to label objects as well as feelings and desires. For example, he may first name a person in order to draw attention and then point and say “look” to the interlocuter to focus on an object He requests information by saying “what” or “what’s that”	He uses simple sentences to direct attention, to request (“can I have that,” “do it again,” “what is that”), and to reject (“I do not want that”) He greets people (hello/goodbye) and communicates a wide range of functions such as expressing himself (“I can do it myself”), naming persons/objects, commenting (“why are you putting it away”), and giving information
Interaction and conversation^c^	He responds to his name and questions directed to him He responds to interactions by looking interested and making eye contact. To join a conversation he may say a/the person’s name, or push his body into another person to get their attention He smiles and laughs, uses clues from the environment and classroom routines to guide his responses He is beginning to use social phrases (“thank you”)	He gains others’ attention and interacts with others via gesture, acknowledging, calling a name, making eye contact, agreeing or disagreeing with others He anticipates, understands/expresses intention, and responds appropriately He initiates verbally (“do you want to…”), maintains, joins (“hey…”) an interaction/conversation; and repairs conversation or requests clarification when necessary; and terminates interactions.

a An independent mother’s pragmatic profile data (age 4.4) concurs with that of the teacher.

b Communicative functions: Requesting, self-expression and self-assertion, and giving information.

c Interaction and conversation: Initiating interaction, conversational breakdown, conversation repair, and joining a conversation.

For example, before the Korean Summer, MJ’s classroom participation often consisted of silent observations of others. His participation was mainly responsive, and conducted primarily through sounds, gestures, eye contact, or the use of one or two words, often imitative (e.g., “what’s that”) when interacting with a teacher or peers. After his Korean Summer, however, his behavior changes in all categories. He initiates interactions, does so verbally, uses full sentences (e.g., “Can I have that?,” “I’m going to do this and you do that”), and comments on what he is doing. He is generally very verbal during play.

In summary, before the Korean Summer, MJ’s language reveals a small yet still developing English lexicon, limited English language use in interactions, a high degree of silent observations, and physical and gestural means of interaction. Most of MJ’s language emerges in the form of one- or two-word responses. After the Korean Summer, he shows much more fluent productivity in his use of English across all pragmatic categories.

### 3.3 General observations (GO)

The systematized transcriptions in GO, given the frequency of assessments (see Figure 2), provide more precise evidence concerning the speed of increase in MJ’s English after his return from Korean Summer and concerning the general nature of the change in his language.

**Figure 4:**
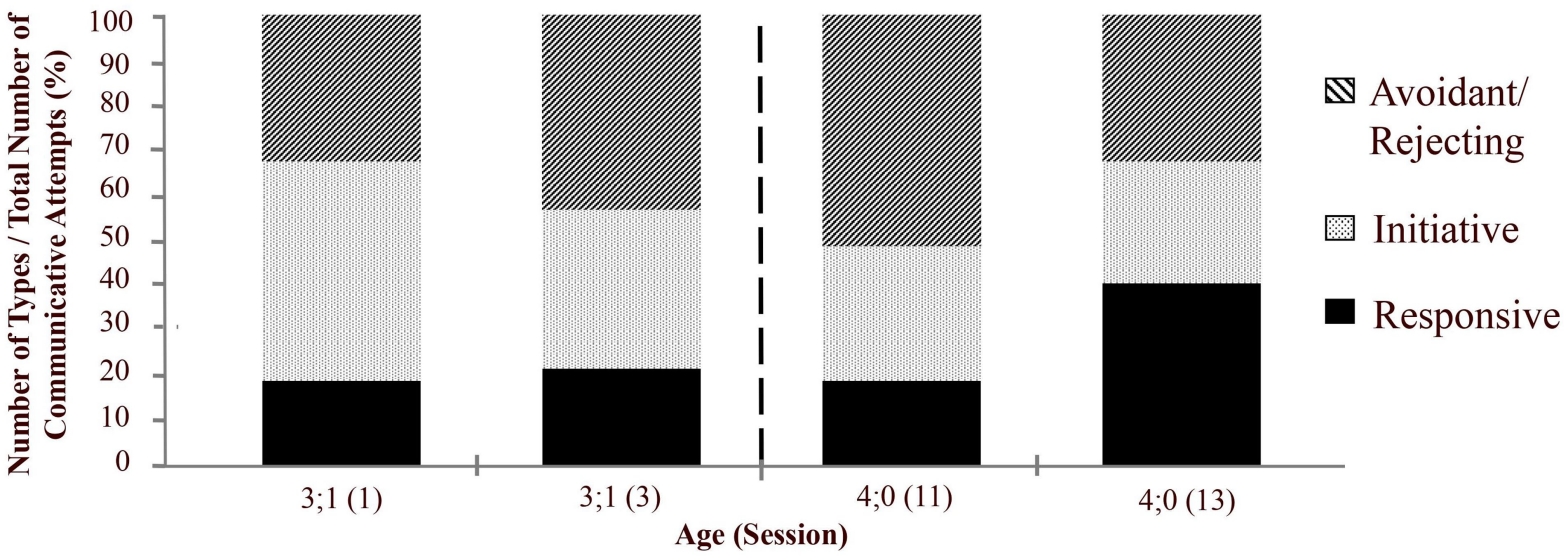
Categorization of three types of pragmatic communicative attempts.

MJ’s attempts at communication in general did not change notably in type over pre- and post-Korean Summer. As seen in Figure 4, “categorization of communicative attempts,” seen in GO, revealed that amounts of initiative, responsive, and avoidance types of communicative behaviors remained consistent before and after the Korean Summer.

**Figure 5:**
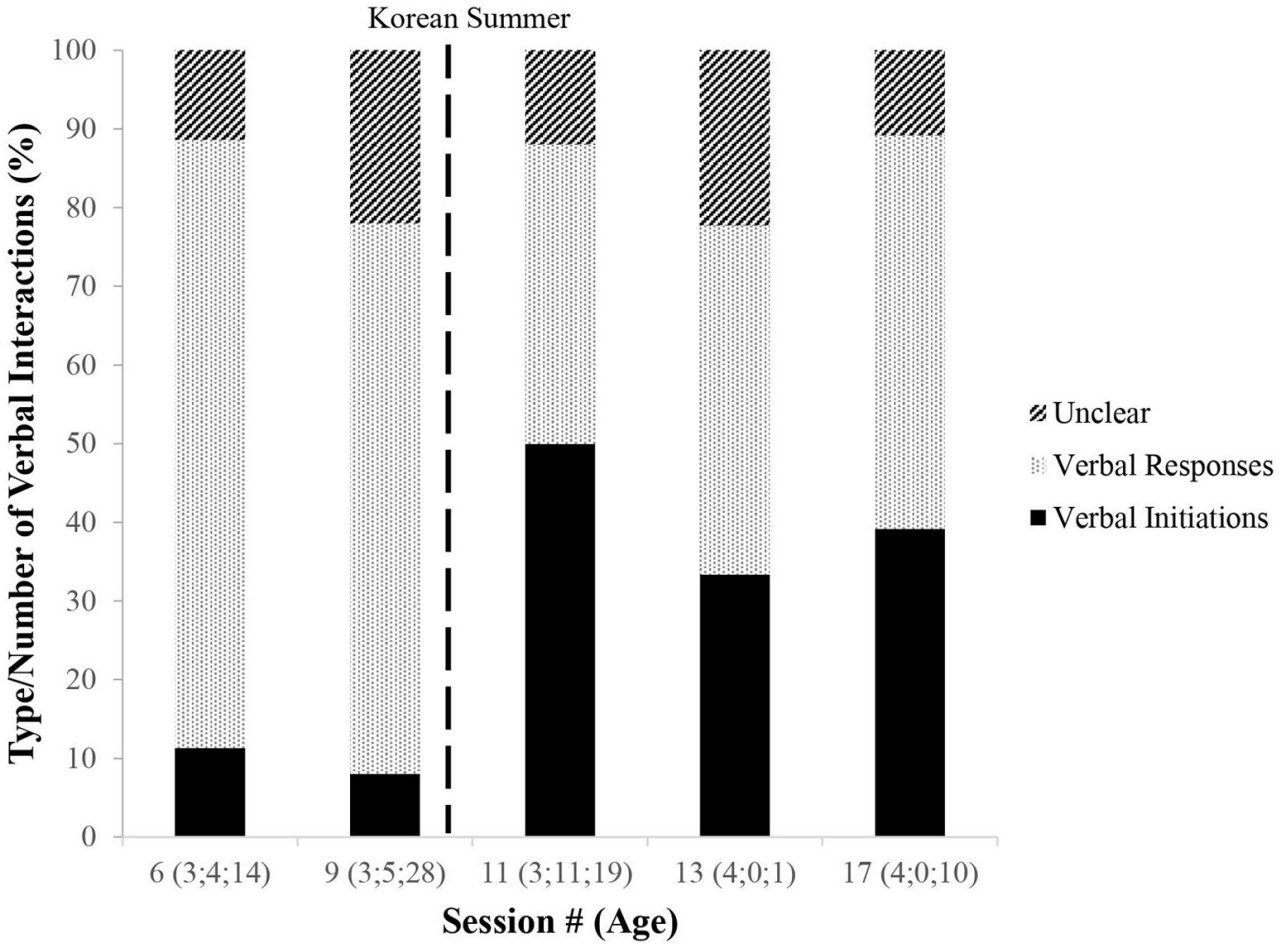
Change in verbal interactions with language.

What did change, however, was the nature of MJ’s language use in pragmatic interactions. When the speech was transcribed during all interactions from the GO reports from pre-Korean Summer early classroom Sessions (6 and 9), analyses were conducted to establish whether MJ’s speech occurred as responsive or as an initiation in interactions. Figure 5 confirms what the GO had revealed. Pre-Korean Summer, MJ’s speech remained mainly responsive, and initiations of interactions that involved language were minimal. However, post-Korean Summer [at Sessions 11 (age 3.11.19), 13 (age 4.0.1), and 17 (4.0.10)], initiations with speech increased remarkably, becoming 50% of MJ’s interactions (Session 11).^12^ In contrast to prominent silence or one-word responsiveness in the classroom pre-Korean Summer, in post-Korean Summer, MJ dramatically increases his use of English in order to initiate social interactions (see natural speech examples in Table 3).

**Table 3:** Example utterances selected from speech samples derived from general observations (GO) pre and post Korean summer (sessions 6, 8, 9 from pre-Korean summer; and sessions 11, 13 from post-Korean summer).

Pre Korean summer			Post Korean summer	
Session 6 (3.4.14; reading a book with experimenter)	Session 8 (3.4.22; playing freely with other children)	Session 9 (3.5.28; MJ with children gathered around teacher in circle; reading a book)	Session 11 (3.11.19; playing freely with other children)	Session 13 (4.0.1; MJ; children playing with blocks; Teacher and students talk with MJ)
MJ: What’s this? MJ: Do not know …. *Experimenter: Where are they going?* MJ: Party MJ: Water *Experimenter: What’s happening* MJ: Those…two people going *Experimenter: What are they doing?* MJ: Not play MJ: I do not know … MJ: More party MJ: That’s a water … *Experimenter: Where are they going?* *MJ: The car. His* car. … MJ: Give me a book	MJ: We(Unclear:are/have) five(Unclear:sets/cents/size), right? … MJ: Do not come in (repeating another child) … Hey, a bug … You said (X Unclear) baby (XX Unclear)	*Teacher: Who could it be?* *MJ:* No answer MJ: I see Iguana MJ: I can see the jaguar, too (repetition of another student) MJ: I want to play now (repetition of another student) …. MJ: Yea, we can do fire, right? … *Student: I can see the pelican and the quail* MJ: And I can see the pe > the quail (repetition) … MJ: It’s not kangaroo	MJ: Let us go in the pirate house MJ: No, it’s not a rocket ship It’s not a rocket ship, right? MJ: Good guy boat faster MJ: Bad guy boat and bad guy boat crashed XXX MJ: We are fighting the bad guy MJ: I’m a prince now MJ: I turn onto the prince MJ: I’m bad guy MJ: I’m not die … MJ: This is a castle. I’m the king of that castle. MJ: Go, bad guy came. Go bad guy store. MJ: Alexander is not doing it MJ: He’s not bad guy MJ: Bad guy boat crash (X) boat. Good guy boat and bad guy boat crash (X) MJ: Princess>princess little beast, and prince is big beast. I’m big beast. MJ” King is>you will be king. You are>you are king and I’m a knight. King is the>king is the strong. Alexander, king is the strongest knight.	MJ: No, I’m not MJ: I’m not playing your game MJ: I do not need that two blocks MJ: You have a lot of them MJ: I’m just two sets MJ: You have a lot of them … *Student: Stop breaking mine* MJ: I’m not breaking your

### 3.4 Natural speech analyses

**Figure 6:**
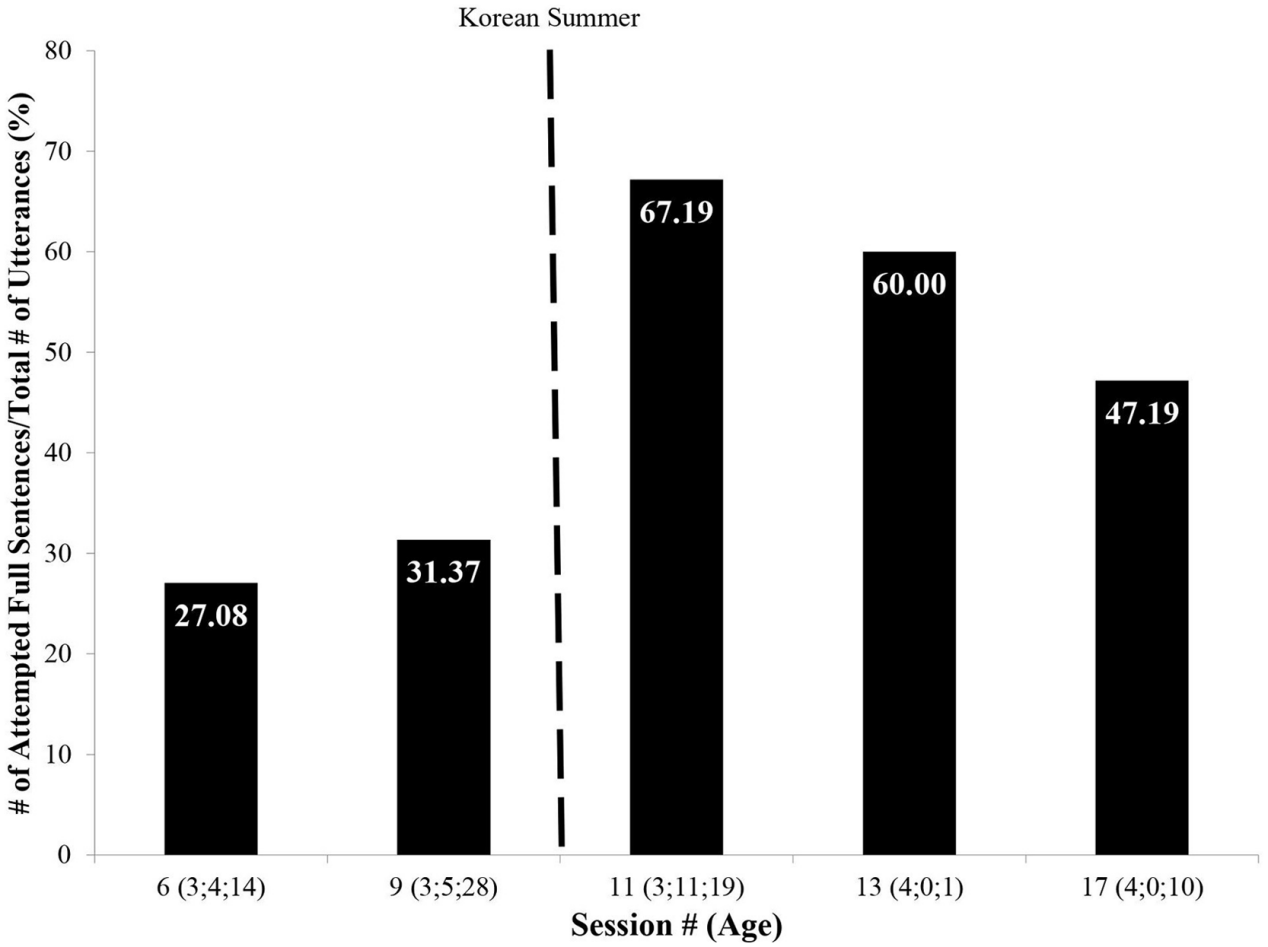
Attempted full sentences.

Analyses of MJ’s speech which were collected during the GO sessions, began to reveal the nature of change in MJ’s language from pre- to post-Korean Summer. As Figure 6 shows and Table 3 exemplifies, in the sessions during MJ’s post-Korean Summer, most of MJ’s utterances were sentences. This is in marked contrast to the pre-Korean Summer sessions where comparable utterances consisted mainly of one or two words.

**Figure 7:**
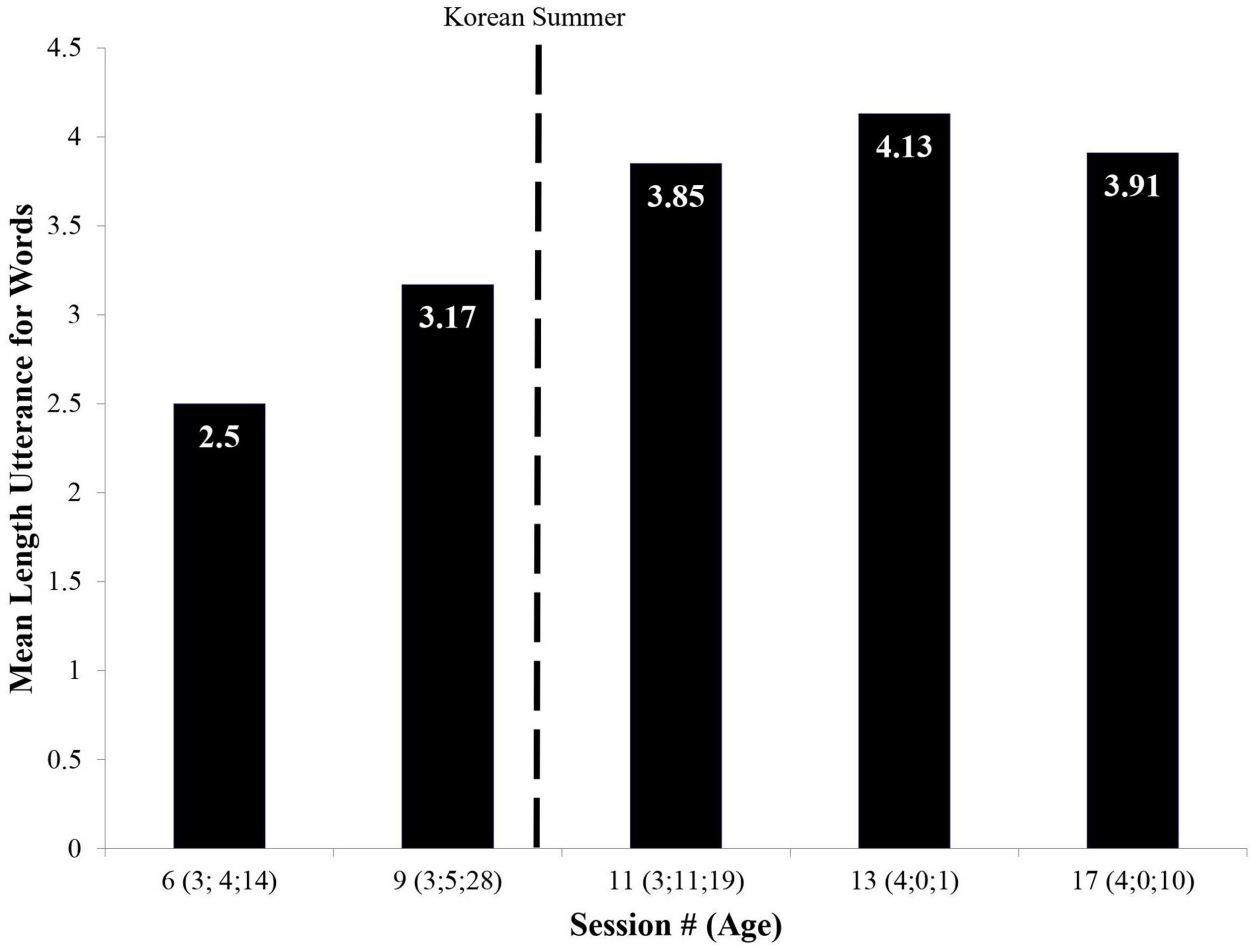
Change in mean length of utterance (MLU).

Analyses confirmed that the linguistic nature of MJ’s attempted sentences notably advanced. As Figure 7 documents, MJ’s mean length of utterance (MLU) increased remarkably from pre- to post-Korean Summer.^13^

**Figure 8:**
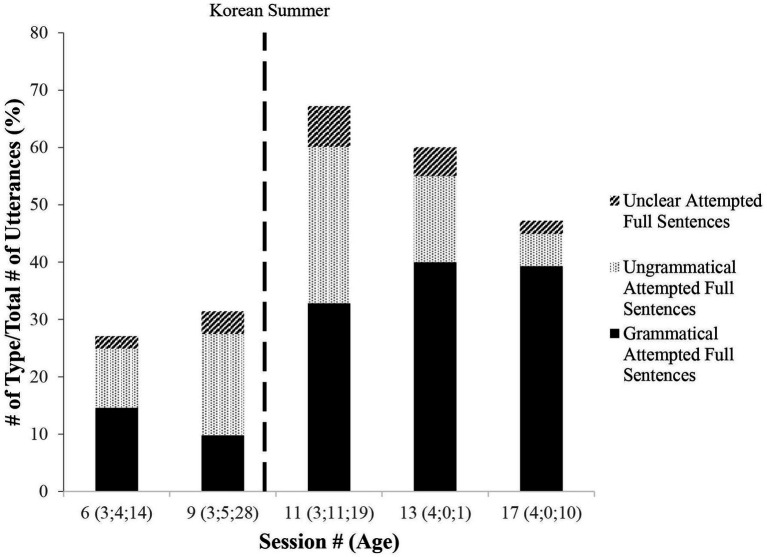
Change in grammaticality of attempted sentences.

As Figure 8 documents, the percentage of well-formed grammatical sentences also increased as a proportion of MJ’s utterances. The *grammatical sentences* were evaluated in terms of aspects of demonstrated English syntax defined prescriptively, e.g., inflection, morphology, and overtness of functional categories.^14^

As Table 3 exemplifies, pre-Korean Summer Sessions 6 and 8 mainly reflect single-word or short word-combinations (*cf.* Supplementary Video S1) as in:

1) Session 6 example

Experimenter: “Where are they going?”

MJ: **“Party.”**

Experimenter: “Are they playing?”

MJ: **“Not Play.”**

In Session 9, just before the Korean Summer, MJ was beginning to construct word combinations at a sentence level, e.g., 2a, often basing these on repetitions of other children’s immediately preceding speech, e.g., 2b. Spontaneous non-repetitions often revealed partially or fully incomprehensible word combinations, and/or evidence of the computational challenge of linguistic integration, e.g., problems in sentential integration of negation in 2c, or of the lexicon in coordination in 2d. Utterances are still only reluctantly inserted into conversations.

2) Session 9 examples.

a) MJ: “**I see Iguana.”**

**“Gimme that.”**

**“It’s not kangaroo.”**

b) classroom student: “I can see the jaguar too.”

MJ: “and **I can see the jaguar too.”**

c) MJ: **“No, you…no [you have two soccer shoes], [soccer].”**

d) MJ: **“Yeah, we can draw fire and PSHH (filler).”**

In contrast, in post-Korean Summer Session 11, we see a change in MJ’s sentence productivity, i.e., the amount of sentence production within the classroom context (as reflected in Figure 6) and in their length (Figure 7). Underlying the length increase, analyses of the natural speech samples reveal developing complexity in combining linguistic operations, e.g., noun phrases, and embedding of these noun phrases in sentence structure, e.g., 3a, and integration of negation in sentence syntax, e.g., 3b.

3) Session 11 examples.

a) MJ: **“[[good guy] boat] faster.”**

**“Go [[bad guy] store].”**

b) MJ: **“I’m [not [knocks it down]].”**

In particular, advances in MJ’s language over the Korean Summer occur even while it is obvious that MJ is still acquiring language-specific properties of English. Language development is proceeding both before and after the Korean Summer. As in examples from Sessions 11 (4a and 4b) and 13, MJ is still developing English inflection post-Korean Summer. An example from Session 13 below shows a mistaken morphological generalization.

4) Session 11 examples.

MJ alternates between:

a) **“I’m not knocking it down.”**

b) **“I’m not knocks it down.”**

5) Session 13 examples.

Classroom student: “Stop breaking mine.”

MJ: “**I’m [not [breaking your]].”**

These productions occur post-Korean Summer, even with the general advances in MJ’s language, which we have seen above. This suggests a fundamentally continuous course of language development before and after data suspension.

Future analyses can help identify the precise aspects of developing grammaticality in MJ’s speech. These analyses can help us to determine more precisely where MJ advances in language during the period corresponding to the suspension of English input and where he does not. What does not advance may reveal where direct input of data may be more significant in the language acquisition process, e.g., language-specific verbal inflection or morphology.^15^

### 3.5 Experimental evidence: elicited imitation

As Table 3 exemplifies, MJ’s natural speech post-Korean Summer productively demonstrates sentential recursion, i.e., coordination, which is critical in the acquisition of syntax and semantics of complex sentence formation. For example, as 6 shows, in Session 11, after his return from Korean Summer, MJ produces:

6) Session 11 examples.

a) “**you are king and I’m a knight,”**

b) **“good buy boat and bad guy boat crash.”**^16^

Experimental evidence regarding MJ’s complex sentence formation through coordination confirms these observations from natural speech; it confirms MJ’s control of essential aspects of coordinate syntax after his return from his Korean Summer. His performance on an elicited imitation (EI) task with factorially designed simple coordination sentences (at Session 26, age 4.1.12) provides this evidence. MJ’s performance on this coordination EI assessment (Session 26) has been studied and reported in detail by Lust et al. (2014).

Results from this EI task offer a detailed analysis of the nature of MJ’s control of coordination and calibration compared to cross-linguistic populations. The EI task replicates an experimental design previously tested with monolingual children (Lust, 1977) as well as other bilingual children (e.g., Lust et al., 2014) and in other languages (e.g., Lust and Wakayama, 1979 in Japanese, Lust and Chien, 1984 in Chinese), in order to assess knowledge of the basic recursive aspect of natural language syntax, which is revealed in coordination, e.g., English ‘and’. On the coordination EI task (Session 26), MJ’s performance on English complex sentences with varied coordination forms was near perfect (81.3%, leaving aside certain errors in inflection, or pronunciation/phonology, e.g., 7a or c, which were not considered failures in the coordination experiment). He dealt easily with all the factors tested in coordinate syntax: sentential and phrasal forms, forward and backward patterns of redundancy, and redundancy reduction in the stimulus sentences. Moreover, MJ’s approach to these sentences resembled that of monolingual English-speaking children, e.g., 7a. The one item he did not imitate accurately was backward phrasal coordination, exactly the type that young monolingual children acquiring English have more trouble with as well, e.g., 7b. He reduced the redundancy in 7c, by transforming it in a forward direction, as do English monolingual children. This example illustrates a syntactic system by which MJ relates two linguistic representations, the expanded and reduced coordinate forms, as monolingual children do (*cf.* Lust, in prep).^17^

7) Session 26 Examples.

a) E: The kitties and the dogs hide.

MJ: **The kitty and the dogs hide.**

b) E: Push and hug the kitty cat.

MJ: **Push and hug and tatty-cat.**

c) E: There are bears and there are dogs.

MJ: **There’s bears and the dog.**

Previous research with monolingual English children (Lust, 1977) reported that children 3 years of age with a high MLU provided 81% correct imitation in this EI experiment. MJ surpasses this. MJ’s performance thus resembles that of children who have mastered the fundamental grammar of coordination in English in a monolingual context.

MJ’s success in the coordination experiment is in stark contrast to the results attained from two other matched young Korean children (CH, YP) learning English in a daycare center in Boston, Massachusetts (See Lust et al., 2014), who were administered the same coordination experiment with EI, but who showed less advanced performance than MJ did, e.g., 8 and 9. These results provide converging evidence supporting MJ’s English language advance.

8) CH; age 4.0.

a. E: Blow bubbles and catch bubbles.

CH: (and) buggles and catch bubbles.

b. E: Push and hug the kitty cat.

CH: Push and kitty cat.

9) YP; age 3.3.

a. E: Kitties hop and kitties run.

YP: Kitties, kitties, kitties run.

b. E: The kitties and the dogs hide.

YP: (and) kitties dog hide.

**Figure 9:**
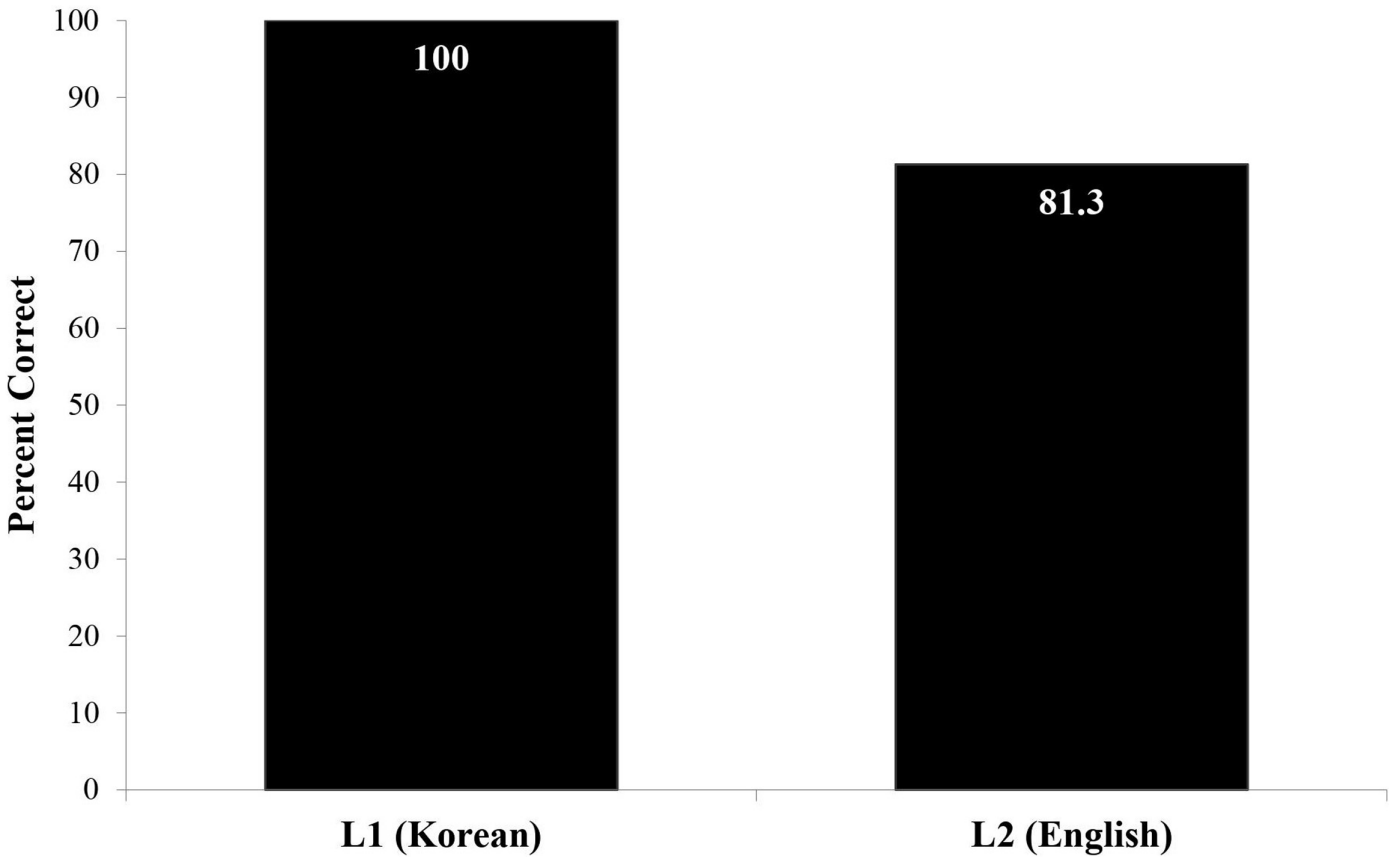
Success in experimental test of elicited imitation in English and Korean: Coordination (Lust et al., 2014).

Although we do not report on MJ’s Korean development in this paper, when MJ was tested on a matched set of coordinate sentences in Korean (See Lust et al., 2014) using a similar design and EI (Session 30, age 4.1.20), he achieved 100% performance. Successful new target (English) language acquisition thus did not require L1 language attrition. This was true despite very basic grammatical distinctions across English and Korean, e.g., word order, and recursion direction, which we introduced above (see Figure 9).

Thus, MJ’s advanced complex sentence formation, which was evidenced in his natural speech immediately after his return from the Korean Summer, is confirmed by experimental evidence (collected about 2 months after his return), suggesting a strong and continuous course of language acquisition despite the interruption of English language input, and converging with the natural speech evidence collected in GO after his return.

## 4 Discussion

In summary, results from this enriched case study reveal that after a period of no input in English (L2), a preschool-aged child has shown no deterioration in his gradual acquisition of this language, but rather significant improvement. This result was revealed through varied assessments which indicated more productive use of English in various pragmatic situations, and development in his language. Evidence revealed that MJ’s MLU advanced, as did his full sentence production and the English grammaticality of these sentences. In general, one- or two-word utterances, or repetitions characterized MJ’s language in his pre-Korean Summer, whereas in post-Korean Summer, MJ’s language revealed fluent and productive sentences in English, including multi-clause recursive structures (coordination) in both natural speech and experimental assessment (i.e., EI). This result complements caretaker’s report by his mother post-Korean Summer.

The results of this case study implicate factors other than direct linguistic input that contributed to this advance, in keeping with our leading Hypothesis 3 (in Section 1.2). We must currently ask more specifically, what then explains MJ’s advancement in English language acquisition in the absence of input from English? We explicate our leading hypothesis in 4.1. We then consider a set of alternative explanations which may appear to explain our results, but in themselves do not. Rather each is compatible with our leading hypothesis.^18^

### 4.1 Our hypothesis: internal integration

*MJ’s grammatical knowledge (of English) advanced during the Korean Summer period without (English) input data: MJ’s prior knowledge coalesced and systematized during the period studied, allowing the advances we have observed over this period.*

In sum, results from this case suggest that language acquisition and language development are not in a direct one-to-one relation with language input. In this, they support Hypothesis 3. MJ advanced in a language (English) during a period in which data from the language being acquired were not available. Only internal cognition could account for the language advance reported here. Thus, the study of the natural Korean Summer intervention has allowed us to see that an essential force of language acquisition lies not directly within the input data, but within the child’s mind. We deduce that internal computation lay behind MJ’s language advance without contemporaneous external data input. MJ began the Korean Summer with some linguistic knowledge of English, although with limited language performance, as indicated above. We suggest that internal computation during the Korean Summer involved the creative integration of this internalized language knowledge. Linguistic integration is not directly determined by synchronic data.

Elsewhere we have argued that this internal linguistic integration is not an occasional accomplishment in language acquisition; rather, it is fundamental and essential throughout the course of language acquisition cross-linguistically. We provide extensive cross-linguistic evidence for this proposal in forthcoming study (Lust et al., in press) through an in-depth study of children’s acquisition of relativization in complex sentence formation. In that study, we argue that linguistic evidence reveals that children across languages integrate universal linguistic principles and constraints relevant to relativization with language-specific facts regarding the language-specific grammar being acquired.

Although we have not studied either MJ’s English or Korean language in detail, early observations that are focused on the relevant natural speech samples (e.g., Table 3) suggest that MJ brings to his acquisition of the new target language, English, certain universal linguistically determined knowledge such as movement or constituent order, e.g., “What’s this,” indicating WH movement, “Give me a book” evidencing VO order of English (Session 6). He does so at the same time that he is still in the process of acquisition of many aspects of the English language-specific grammar. For example, post-Korean summer (Session 11), as we saw above, he fluctuates in the use of functional categories such as determiners in “It’s not kangaroo,” as is common in first- (*cf.* Valian, 2024) and second-language acquisition (Kedar, 2018). Despite productive sentential coordination, speech samples post-Korean summer reflect an absence of English-headed relativization, resembling early periods of syntax in language acquisition (*cf.* Lust et al., in press), e.g., “King is the…king is the strong…” (*cf.* [The king is [[the one] who is strongest]]). We have argued (in Lust et al., in press) that the creation of a linguistic system in the language being acquired is a fundamental property of this internal computation. In independent research, Lakshmanan (in press) has provided evidence that the creation of a linguistic system is critical not only in acquisition but also in the maintenance of an acquired language. (See Lakshmanan, 2009 for the call to “study the role of internal procedural mechanisms that motivate a change in the mental representation…” of the child during language acquisition (p. 384).) Further research can investigate how universal and language-specific linguistic factors interact in this process.

Given that MJ was not receiving input data in English (L2) during his Korean Summer, but also not producing it,^19^ our results may also pave the way for an understanding of a previously unexplained but reported finding of “silence as a consistent and typical characteristic of childhood second language acquisition” (Roberts, 2014, p. 22; Lakshmanan, 2009).

In particular, our analyses of the development of language during an input-deprived period involved several linguistic dimensions of language knowledge. It involved grammatical knowledge, as analyses of sentence grammaticality, length, and language-specific inflection shown above. In addition, there was development in MJ’s language use (e.g., language productivity and pragmatic interaction) (Table 2). We speculate that increased integration of linguistic knowledge, resulting in increased systematicity would have enabled increased pragmatic facility, thus explaining their joint development. We would reasonably expect that advances in systematicity in linguistic computation would have the effect of increased fluency and automaticity in language use.

The ultimate question remains: *if linguistic integration underlies advancement in language acquisition in the absence of synchronous data input in that language, what is the nature and content of such linguistic integration?*

### 4.2 Alternative explanations for MJ’s advance

#### 4.2.1 Alternative 1: Korean input

*During the Korean Summer, MJ was not denied exposure to all language. He was exposed to a continuous amount of Korean input and interaction. Korean being the only language MJ was exposed to during this period, we can ask: does the mere fact of language exposure in one language increase capacity in another language, even without data from this other language? The first language (Korean) is developing during this time; does this explain the development of the new target language (English)?*

While we do not assess MJ’s Korean L1 language acquisition in detail in this paper, it is clear that his Korean is well developed at the beginning of his ECC experience and becomes more so over time. When comparisons are made of MJ’s production of coordination to those of a control English–Korean bilingual child of a similar age, as in Lust et al. (2014), MJ is superior in both his Korean and English. These findings confirm that MJ’s Korean was and remains strong over the period he was studied.^20^

While this factor of the strong and continuous first language (Korean) may play a role in explaining our findings (e.g., Meisel’s, 1990 ‘mutual reinforcement’ proposal), it does not directly account for them. Although the relation between two or more languages during development remains to be explored, here we see that it cannot be direct, e.g., through some form of “direct transfer” from one language to the other. As we noted above, Korean and English differ syntactically, e.g., in branching direction and head direction, including verb final order in one language, verb medial in the other as well as in all the deductive consequences which follow from this variation: morphology, phonology, case marking, and inflection differ as well. MJ’s highly successful EI production of coordinate sentences in English (L2) required him to assess and systematize multiple different factors across this variation, in contrast to those in his first language. Whatever mutually enforcing relation might exist during the acquisition of two languages it must be indirect, and given the Korean Summer, it would have had to occur independently of direct English data input. If so, the mechanisms for this indirect data-independent influence of one language on another remain to be explained.

In future research, we can currently ask whether and how linguistic systematization in one language may advance systematization in another language. In early study (e.g., Meisel, 1990), it is proposed that there is evidence that “bilinguals succeed more easily than monolinguals in decoding the language-specific coding systems and the underlying grammatical principles” in language (p. 18), reflecting the possibility for a type of “mutual reinforcement” between languages in language acquisition. Flynn et al. (2004) provide a research program pursuing this general issue. They report findings regarding specific principles of cross-language integration and test their proposal in the *Cumulative Enhancement Model*; i.e., all “known” language grammars can contribute to the subsequent development of new target-specific grammars. Their program seeks to determine how and when this occurs.^21^ Flynn et al. (2004) provide evidence from research on the effects of specific grammatical variation among languages during multiple language acquisition, revealed through studies of the acquisition of a third language, given various defined linguistic forms of a second language.

If the continuous experience of Korean does underlie MJ’s advances post-Korean Summer, then universal principles of grammar may underlie the internal integration of a new specific language system, even while new specific language data input is suspended. In this case, this alternative explanation is compatible with our hypothesis of internal integration and provides direction for its further study.

#### 4.2.2 Alternative 2: pragmatic development

*The child’s pragmatic competence developed during the Korean Summer months. Perhaps the child’s language development is directly explained by this pragmatic development.*

Again, these facts alone do not explain our results. They may explain in general why the child has become energetically more productive with language, e.g., initiating verbal forms of communication more frequently. However, such developments alone could not have led to the specific linguistic advances we have observed. In fact, many of the social pragmatic behaviors of the child in this study are evident before and after the Korean Summer (see Table 2 and Figure 4). What changes in the linguistic content of MJ’s language and its use? In several ways, MJ’s pragmatics did not develop; for example, he was and remained communicative and socially willing to communicate. As seen in Table 2 and Figure 4, general forms of communicative attempts did not develop noticeably over 1 year.

The increase in pragmatic fluency in MJ’s language itself must be explained. Advances in internal linguistic integration in MJ’s language (our hypothesis) may explain the increased fluency in production in MJ’s pragmatic functioning with language, e.g., his increased use of sentences to initiate interactions. We would expect systematicity in grammatical knowledge to facilitate fluency in production and thus assist social interactions. In fact, we have argued elsewhere that it appears that the child’s syntax was being acquired at a faster rate over time than either his vocabulary or his pragmatic development (Lee et al., 2007).^22^^,^^23^

#### 4.2.3 Alternative 3: production develops

*During pre-Korean Summer, the child knew more than he produced. What developed was his production, not any aspect of his grammatical knowledge, during the period studied.*

This suggestion relates to, but is not identical to, our own hypothesis. In fact, when one looks more closely at the data suggesting a change in MJ’s language, e.g., in Table 3 examples, it appears that frequent *omission* of functional morphemic elements characterizes the pre-Korean Summer periods, e.g., 10.

10) “**the car”** [to the car].

“**It’s not kangaroo.**” [It’s not a kangaroo.]

**“I see Iguana.”** [I see an Iguana.]

If this is the case in general, then null elements in the earlier period may have simply underestimated the child’s grammatical knowledge. In fact, a wide array of studies of monolingual first language acquisition have provided evidence that children access functional categories before their language production, even at younger ages including infancy (e.g., Kedar et al., 2017; Valian et al., 2009; Valian, 2013; Yang and Valian, in prep; Dye et al., 2019 and Shi, 2014 for review). Then, could the ‘developmental’ or ‘acquisition’ change that has occurred in MJ be explained simply by a phonetic and phonological realization of existing grammatical knowledge expressed through overt production post-Korean Summer?

Access to prior knowledge is surely involved in the grammatical integration which we hypothesize characterizes MJ’s advancement. We do see in post-Korean Session 11 and thereafter, for example, the more productive^24^ phonetic realization of previously null forms of functional categories, such as auxiliaries, determiners, various forms of inflection, and prepositions, as well as the realization of lexical arguments such as sentential subjects and objects, e.g., 11a. Although various forms of language-specific inflection are still developing in Session 11 and beyond, as can be seen in the irregularity of examples in 11 below and Table 3, many of these are being realized in Session 11 overtly in a way they were not productively, in the earlier sessions (pre-Korean Summer). Thus, descriptively, it appears that changes in MJ’s language over time include the productive realization of grammatical functions, which may have been simply phonetically null in preceding sessions, although he is still learning the language-specific grammar of these forms. (See Kedar, 2018 for a study of the course of determiner acquisition in a bilingual Hebrew–English situation.)

11) Session 11 Examples.

a) “**I’m a prince now.”** (in game).

**“I turn onto the prince.”**

b) “**I’m bad guy.”**

“**I’m not die.”** (in game).

c) “**I’m not knocking it down**.”

**“I’m not knocks it down.”**

However, MJ’s language post-Korean Summer makes clear that his development is not simply a matter of the development of speech production, i.e., simple insertion of overt functional forms, but of his grammatical integration of knowledge. For example, MJ’s alternation post-Korean Summer between “I’m not knocking it down” and “I’m not knocks it down” (Session 11) in 11c (same as 4 above) reflects an ongoing still wavering exploitation of English verbal inflection, as well as of sentence integration of ‘not’/negation. “I’m not die” reflects an ongoing acquisition of the English auxiliary system [I do not die; (in this game)]. “He’s not bad guy” reflects an ongoing acquisition of a complex determiner system that cannot be determined simply by insertion, [a bad guy/the bad guy].

We saw that coordination in MJ’s language pre-Korean Summer involved an indication of some early coordination knowledge, e.g., 2d, but it showed increased systematization in post-Korean Summer. MJ’s fluent control of coordination in the elicited imitation experiment confirms the grammatical integration of syntax and semantics which go beyond the overt production of functional categories, as does his post-Korean Summer natural speech, e.g., 12. In general, the sentential-level advancements in MJ’s language pre- and post-Korean Summer do not simply depend on the realization of functional categories.

12) Session 11 Examples.

“Good guy boat and bad guy boat crash” (XXX = unclear).

Accordingly, while the short MLU in pre-Korean Summer (Session 9) (MLU 3.17) includes an upper bound of 6–8 words, this is primarily in repetitions of other students, or of himself, e.g., 13.

13) “I can see XX octopus” (XX = unclear).

“And I can see the octopus XX.”

In post-Korean Summer (Session 11), the upper bound of language (MLU 3.85, UB 8–9 words) is reflected in the systematic productivity of full sentences allowed by syntactic coordination, as in 12 or 14.

14) “You are…you are king and I’m a knight.”

Coalescence of grammatical knowledge, i.e., linguistic integration, may be necessary for the learner to realize phonetic knowledge in language production, including that of functional categories, or to realize the increasing length of utterances. This productivity would surely exploit prior knowledge where it does exist. However, in contrast to speech production of functional categories as a cause of observed language advances, it may be linguistic integration of general linguistic knowledge which underlies growth in functional category production and general speech (e.g., MLU).

### 4.3 Limitations and future directions

#### 4.3.1 Limitations

The specific investigation we have reported here, even though reflecting a systematic infrastructure (see Figure 2 and summary in our Appendix), cannot be exactly replicated, as with any ‘natural experiment’, or any case study. As we noted above, with the *enriched case study method*, the conduct of the research, the data available at any time, e.g., the choice of assessments administered, the qualities of the child at any time, and the general environment at any time, cannot be precisely pre-ordained. (See also Valian, 2021 on individual variability in behavioral data.) In the end, the method rests on the child, who each day and time, may or may not be willing and ready to participate in systematic investigations, e.g., specific tests such as the PPVT or an EI experiment. The method rests on a strong relationship between the researcher and the child which can be maintained over time and wherein experimental assessments can be inserted naturally with general observations.^25^ It requires sustained investigation over time, thus raising the well-known challenges of longitudinal research. Thus, the full program of assessments designed in the enriched case study method is in itself an idealization. Each resulting individual study reflects a selective set of assessments conducted for a single child.

#### 4.3.2 Future directions

What can be replicated is the blueprint for investigation that we have established, one which can result in a rich data set over time, allowing unlimited new research questions.

In particular, although pre-planning for natural events over time cannot be pre-ordained, the instance of young second language learners in nursery school immersions, who change environments for a natural summer break, or other cross-cultural visits, may be an under-tapped population for further research study. Now that the ‘natural experiment’ of summer intervention in nursery school situations has provided significant evidence of the nature of language acquisition, specifically with regard to the role of input data, additional research with additional children can specifically pursue and design timing of before–after investigations of such cases, as well as other potential variables.

The Multilingualism Questionnaire can provide a backbone for such investigations, as can structure general observations, and the integration of existing experimental designs and methods to evaluate critical aspects of language acquisition where monolingual comparisons are available (e.g., Lust et al., 2014; Kim et al., 2018). Selected monolingual comparisons provide critical calibration with a general population and potential for generalization of case study results, as can integration of standardized tests at different time intervals. Systematic comparison across case studies involving different languages can help dissociate languages and language experience within the bilingual or multilingual individual.

For example, in general, even an irregular collection of systematically designed data points can and does lead to significant discoveries regarding language acquisition, e.g., comparison of direct experimental assessments to parent/caretaker questionnaire data (e.g., Lust et al., 2014), comparison of growth across different dimensions of language acquisition, e.g., syntax vs. pragmatics or vocabulary vs. syntax or pragmatics (Kim et al., 2007, 2018; Jeon et al., 2008; Lee et al., 2007; Park et al., 2006), comparison across first and second/or multiple languages, evaluation of language attrition (e.g., Lee et al., 2011), and other issues. Even more generally, repeated assessments over time, within and across language dimensions, can inform a limitless number of hypotheses regarding the nature of language development. (See Appendix for a link to complete raw data archive.)

The infrastructure of naturalistic, standardized, and experimental tests, which we have adopted (Figure 2, Appendix), can be extended and tailored to particular research interests. In particular, our infrastructure has depended on language production to a large degree. We have argued that production data reveal linguistic knowledge more directly than comprehension data does.^26^ However, the issue of how the child’s comprehension is developing when production is not manifest remains a critical and a fascinating one. In future research, further tests of language comprehension can be added to a longitudinal suite of tests.^27^

Finally, what can be replicated, and what is necessary for research advancement in the general field of language acquisition is a systematic and standardized methodology for data collection, recording, and archiving (e.g., Blume and Lust, 2012, Blume and Lust, 2017; Blume et al., 2019a; Blume et al., 2019b; Pareja-Lora et al., 2019); one which can be expanded to multilingual and developmental data. University Research Library support is critical for the preservation of data and systematization of archiving metadata rendering data findable and reusable (e.g., Words of the World’s Children special collection at Cornell University Library^28^) (see Rieger, 2019).

#### 4.3.3 New research questions

Our specific results in this paper provoke new more specific research questions which can be pursued based on our methodology and on future data archives such as ours. For example, how can we assess the developmental trajectory of language acquisition as it is affected by data suspension and the mental computation that it involves? In what ways is this course continuous or discontinuous?^29^ More specifically, we have seen (e.g., Section 4.2) that MJ’s language post-Korean Summer was still developing in language-specific features of inflection and morphology, while maintaining and exploiting certain universal properties of language, e.g., coordinate syntax. Further specific analyses of language pre- and post-suspension, and further data across other cases of suspension can target this dissociation more specifically. To what degree are universal linguistic properties continuous, while language-specific ones are not? Exactly what constitutes language advances when and where they occur in the absence of data input. Finally, and perhaps most fundamentally, how can we discover the nature of internal mental computation leading to grammatical integration?

## 5 Conclusion

Our results provide evidence that in language acquisition, the role of external data input is more indirect than often assumed. Language data input does not directly determine language development. Our research results provide insight into the nature of language acquisition in general, monolingual or multilingual. The case of second language acquisition studied here provides a window into the nature of language acquisition and a unique opportunity for its empirical study.

The most significant consequences of our research are twofold. They lie not in any particular result, but (i) more fundamentally in re-orienting the study of language acquisition to a concentration on and study of, not merely the input data to which children are exposed, but on the child’s mind and the internal computation which the child’s mind silently, and tacitly conducts independent of direct input, in order to create a language, either one language, or more than one. (ii) This research drafts the outlines for a methodology for the scientific study of this elusive but critical dimension of language acquisition, a methodology which can currently be refined. The combination of focused experimental methods with naturalistic observations, embedded in a systematic archival infrastructure, allows us to test hypotheses and enriches the significance of comparable naturalistic observations in turn.

## Data Availability

Publicly available datasets were analyzed in this study. This data can be found here: https://rmc.library.cornell.edu/EAD/htmldocs/RMM08525.html.

## Footnotes

^1^Our records are dated in accord with the 49 sessions in which we studied MJ. Because we did not know the significance of the Korean Summer event, we do not have the exact dates of MJ’s travel.

^2^An MQ (by MJ’s mother) reveals that prior to his entry to the English language nursery school MJ had previously visited the U.S. between 7 months and 1 year 11 months of age. During this time, MJ had infrequent and irregular exposure to English through a day care center he attended for several hours a day several days a week.

^3^For introductory study of case assignment in Korean, see Lee (1991); for introductory experimental study of Korean-English comparison in language acquisition see Lust et al. (2014), Lee et al. (1990); for study of acquisition of Korean, see Kim (1997), Choi (1997).

^4^During the period of this study, researchers in the Cornell Language Acquisition Lab were pursuing an overarching general research question: how in the first few years in an English only nursery school does a child successfully acquire English as a new target language?

^5^Our method here shares in part with the approach developed by Scholz and Tietje (2002).

^6^The method itself is open-ended, thus allowing any researcher to modify the set of linguistic components, e.g., to include morphological or phonological testing (especially where standardized calibration is possible).

^7^We apply this method in this paper to a child acquiring a second language; the method *a priori* is, of course, applicable to monolingual children as well.

^8^Session 6 involves a one-on-one session with the researcher, Session 9 involves a group situation in the classroom with the teacher.

^9^In accord with the method sketched in Figure 2, MJ’s complete assessments include some in Korean as well as in English. We do not include their analyses in this paper.

^10^Although we focus on recordings more closely related to the dates of the Korean Summer, we consider other data along the timeline of 49 sessions to be also critical, i.e., they bear on the nature of the ‘course of development’ in language acquisition and the impact of input suspension on that course. Although we cannot assess the full ‘course of development’ in this child In this paper, we base our evidence on sessions critical to our argument, where raw data have been transcribed, reliability verified, analyzed and systematically archived according to standardized formats. See Appendix for location of full set of archived raw data across 49 sessions.

^11^ https://testvcla.cac.cornell.edu/the-vll/the-multilingualism-questionnaire/

^12^Note that especially in Sessions 13 and 17 when speech now reflects ongoing conversations, it is difficult to differentiate ‘initiation’ from ‘response’.

^13^MLU is generally interpreted as a general, although limited, measure of language development (see Blume and Lust, 2017 on MLU on the complexities and limitations of MLU as a measure of grammatical development). General ‘grammaticality’ is also only a general and limited measure of grammatical development. Further linguistic analysis of sentence structure within utterance will be necessary to characterize more precisely the nature of the advance in grammaticality which MJ’s speech reveals.

^14^For example, in 4 below, although “I’m not knocks it down” was scored as ‘ungrammatical’, “I’m not knocking it down” was scored as ‘grammatical’.

^15^These facts cohere with a Grammatical Mapping (Lust, 2012; Lust et al., in press) paradigm for study of language acquisition. In this paradigm basic principles of Universal Grammar are recognized to be continuously available during development, while language-specific grammatical factors develop over time through UG guided experience. Notably MJ’s pre Korean Summer speech did reveal early restricted coordination use. E.g., as sentential discourse operator in his repetition in Session 9 (Student: I can see the octopus > MJ: And I can see the octopus XX.”) or in his attempted phrasal coordination (see in Session 9, example 2d above).

^16^It has long been noted that recursion first appears in natural speech after a MLU of 3.5 is reached (Brown, 1973), probably an effect of overcoming the universal length constraint on early productions (Lust, 2006).

^17^E = experimenter.

^18^MJ had returned to the English nursery school post Korean summer in early fall; our first post assessment for English data was Session 11. Could the child’s advances have been due to the child’s experience during the first few days of his return? However, even if the child is surely using these first days of renewed English experience, it does not seem to provide an explanation for the complex and continuous advances we witness. The question would remain as to why language experience had such a quick effect during these first few days, as opposed to the slow progression over the preceding 12 plus months of the child’s continual English exposure; and as to how the systematicity of language advances could be so explained.

^19^MJs mother reports that he spoke Korean on the flight home for the summer, and spontaneously switched to English on the way back to the US. There was no English input during the summer.

^20^These results also provide evidence regarding the well studied issue of L1 attrition during L2 acquisition. MJ showed no attrition of Korean complex sentence formation (coordination) and related syntax during his new target language English acquisition during the period studied here.

^21^Further study of this area may reveal distinct processes of linguistic integration (in accord with our hypothesis). *Cf.* Flynn et al. (2004).

^22^For the study of the subtleties of language-specific pragmatic development when considered in detail beyond the broad categories we have considered here, see for example Blume (2021).

^23^Another potentially related more general hypothesis is possible: *Some form of “maturation” explains the observed language acquisition.* Although some form of biological maturation did occur in this typically developing child, and this too must surely be involved in a full explanation of our results, this explanation is not sufficient either. There is no definition of what the term “maturation” means in this case, and no viable explanation for how English grammatical knowledge ‘matures’, or what this would mean biologically. (For example, the specific-language linguistic advances discussed above.) (*cf.* Lust, 1999). “Maturation” in this case is what is to be explained.

^24^Note the ambiguity in the word ‘productivity’ here. While language ‘production’ which we consider in this hypothesis refers to the mechanism of overt speech (or sign in the case of sign language), ‘productivity’ in the language data we consider here refers to the regularity and frequency of the occurrence of particular linguistic phenomena; e.g., overt determiners. Thus ‘productivity’ is a property of and reflection of the system lying behind language production.

^25^Research reported in this study, resulted from work in the Cornell Early Childhood Center (ECC) which existed through the Department of Human Development and the College of Human Ecology for several years. Led by director Elizabeth Stilwell, students both undergraduate and graduate were taught to interact with children and the center and allowed to become part of the regular environment of staff and children and to do so over time. ECC teachers became co-investigators, providing general observations. The ECC was terminated in 2008, a year after our last interview with MJ. Our research also depended on a superb collection of students (many of them undergraduates) from the Cornell Language Acquisition Lab (CLAL) interested in working with both child and a teamed research endeavor, either continually over years, or one replacing the other over time; all committed to learning basic techniques of archiving shared data. For each case, students bilingual in the language of the case participated. And of course, without the child, MJ, our results would not exist.

^26^See Blume and Lust (2017) for a review of both comprehension and production methods and the quality of converging evidence across these (also Lust et al., 1987; Dye and Foley, 2020). See Menenti et al. (2011) for a study of the neural basis of comprehension-production relations.

^27^As one reviewer suggested, “It would be great to know what was the child’s comprehension abilities in English before and after their Korean summer.”

^28^ https://rmc.library.cornell.edu/EAD/htmldocs/RMM08525.html

^29^This question was suggested by a reviewer.
