# Supplementary material for: Acquisition of a new language: an enriched case study documents language growth without external input in a young Korean child’s acquisition of English
Source: Front Hum Neurosci. 2024 Nov 27;18:1456054. doi: 10.3389/fnhum.2024.1456054 (PMC11631620; doi:10.3389/fnhum.2024.1456054)
Supplement: Supplementary file 1 [file Data_Sheet_1.PDF]

Virtual Linguistics Lab

Child Multilingualism Questionnaire[1]

Purpose: The purpose of this questionnaire is to gather background information on children who are bilingual, multilingual, or have been exposed to a bilingual or multilingual language environment. Through this information, the parents/caregivers, teachers, and researchers can come to a better understanding of the nature, quality, and degree of a child's developing bilingual/multilingual language acquisition. This questionnaire is divided into six sections shown in the contents overview here.

<u>Contents</u>

Administrative Information & Instructions to the Interviewer

Participant & Interviewer Information

Part I. Information about the Child

Part II. Child Language Information

Part III. Family Background Information

Part IV. Code-Switching (Language Mixing)

Part V. Reading/Writing

---

[1] Prepared by the Cornell Language Acquisition Lab and Virtual Center for Language Acquisition Members and affiliates in 2015.

Part VI. Comments

Supporting Material I – Child Weekly Activities

Supporting Material II – Proficiency Scale for Oral Comprehension (Listening)

Supporting Material III – Researcher’s Judgment on Child’s Language Proficiency

History of the VLL Child Multilingual Questionnaire

The initial stages of this questionnaire grew out of lab meetings at the Cornell Language Acquisition Lab (CLAL), where a group was conducting experimental research on the acquisition of English in monolingual children. Many subjects were characterized as ‘bilingual’ by teachers’ report, which would eliminate them from the study. The lab members saw the need to develop a more systematic way of capturing the nature, quality, and degree of bilingualism and multilingualism in children. This concern grew even more important when a new research group, led by then graduate student Sujin Yang, began to investigate early childhood multilingualism.

At that time, the Bilingual Questionnaire by Weber-Fox and Neville (1996) was consulted. As this was aimed at older children and/or adults, it was realized that a questionnaire geared towards younger children was needed. Group members constructed the CLAL Multilingual Questionnaire for Parents. It was then circulated to the founding members of the Virtual Center for the Study of Language Acquisition for input and revised as the “Virtual Linguistic Lab Multilingual

Questionnaire for Parents.” In particular, Professor María Blume (then at University of Texas at El Paso) integrated it with another questionnaire that Professor Liliana Sánchez (Rutgers University) had been developing specifically for the study of multilingualism.

CLAL members, who participated in constructing the initial form of the questionnaire and administering it to parents, included: undergraduate students Anne Spillane, Michelle Cerny, Sarah Burger, Amy Tsai, Kari Schaap, JoAnn Shih, and Michael Natanzo, as well as then graduate students Sujin Yang and Yarden Kedar. Sujin Yang assumed major responsibility for developing the first form of this questionnaire. The first version of the questionnaire, which incorporated VLL member input, was developed by María Blume, Anne Spillane and Barbara Lust in 2003. An expanded revision was developed by the Multilingual Questionnaire project team in 2004–2006: Michael Natanzon and Sujin Yang, with the help of participants in the Cognitive Studies Research Workshop, Fall 2005. The following people were involved in a 2012 revision: undergraduate students Nicolas Champagne-Williamson and Steven Lin, graduate student Carissa Kang, and research assistant Seong Won Park. Seong Won Park provided important edits and she created a database to collect completed MQs. (Available from www.clal.cornell.edu/vcla). 236 filled MQs which include English and Spanish as well as Chinese language versions, were collected from Cornell University (CLAL and Qi Wang Lab), MIT, and University of Texas at El Paso at that time (May 15, 2007). A first scoring template by

which to summarize data from each MQ and evaluate a child's overall bilingual/multilingual proficiency was created by Seong Won Park. Analyses on these collected filled MQs are being conducted through this evaluation form to assess scoring reliability. The Standardization and Quantification of Lingualism (SAQL) group at Cornell created a short version of the existing questionnaire in Spring 2011 and further revised it in Spring 2012, readying it for reliability testing against the long form. This short form now exists as a highlighted version of this MQ (and is independently available). An adult version is under development.

In 2006, the members of the University of Texas at El Paso (UTEP) Language Acquisition and Linguistics Research Lab (LALR lab) started to work on a Spanish version of the questionnaire. The students, who then under Ellen H. Courtney and María Blume's direction, created and tested for the first time this version of the questionnaire were: Elsa Aguilar, Luis Caballero, Félix Fernández, Martha Domínguez, Jennifer Manssour and Raquel Salazar.

A second revision of both the adult and child questionnaires in their English and Spanish versions was undertaken by the members of the LALR lab in the Spring 2010. Contributors to the questionnaire were Dr. Ellen H. Courtney, Dr. Alfredo Urzúa, Dr. María Blume, graduate students: Brannon Bradford, Félix Fernández, Cliff Jones, Marina Kalashnikova, Jaime Ontiveros, Martha Rayas, and undergraduate students: Laura Chávez, Abraham Jallad, and Harat Saucedo. In Fall 2010, the undergraduate students Mayra Larios, Raquel González and Lilian

Ávila contributed to the revision. In Spring 2011, the undergraduate students María Jiménez and Martha Rayas continued with the revision of this questionnaire.

Researchers in the Cornell Language Acquisition Lab, led by Carissa Kang and Alicia Kim have now begun an analysis of MQ results based on a research study of bilingual children in Singapore directed by Carissa Kang.

Thanks to multiple contributions by Virtual Center for Language Acquisition members, the current MQ (2015) has undergone numerous revisions since its first form in 2003; and further revisions are anticipated as the instrument is tested for use.

Comments on possible improvements to the questionnaire are welcome. Please contact Professor Barbara Lust (bcl4@cornell.edu) and Professor María Blume (mblume@pucp.pe) with any questions or comments.

Other Language Versions of this MQ which are under development include Spanish and Korean.

Founding Members of the Virtual Center for the Study of Language Acquisition (VCLA): http://www.clal.cornell.edu/VCLA/index.html who have also contributed to the development of this questionnaire include

Pontificia Universidad Católica del Perú- *María Blume*;

Rutgers University at Newark - *Jennifer Austin*;

California State University at San Bernardino - *Yuchin Chien*;

MIT - *Suzanne Flynn*;

Boston College - *Claire Foley*;

Southern Illinois University at Carbondale - *Usha Lakshmanan*;

Rutgers University at New Brunswick - *Liliana Sánchez*;

Cornell University - *Barbara Lust, Marianella Casasola, Qi Wang; and*

Nielsen NeuroFocus Group - *Elise Temple.*

Administrative Information

This questionnaire should be administered to parents or caregivers in collaboration with an interviewer. Where sections or questions are not relevant, simply leave blank or write 'NA' (Not Applicable). **In the first stage**: Parents or caregivers may complete the questionnaire at home and return it to the school director or teachers; the interviewer may work with the parent or caregiver to complete it. The latter is preferable, if possible. If you are unable to complete the whole questionnaire at this time, please just answer the highlighted questions, which constitute the short form. **In the second stage**: Once the questionnaire has been returned, the interviewer will conduct an interview either in person or over the phone to complete the fields that need further clarification or explanation.

Instructions to the Interviewer

Please send a copy of the completed questionnaire and evaluation form to __(head researcher/group)__. They will be entered into the emerging cross-linguistic database.

Replace subject and caretaker names with Subject IDs (first and last initials plus birth date (mmddyy, MB012468 for example)) before sending the document. Keep the original copy with the identifying subject information confidentially for your own records.

Participant & Interviewer Information		
P1. Participation Date	(mm/dd/yyyy)	
P2. Parent/Caregiver Information	Name:	
	Phone:	Email:
	Relation to the child:	
P3. Interviewer (Research Assistant) Information	Name:	
	Phone:	Email:
P4. Person Completing the Questionnaire	☐ Interview ☐ Parent/Caregiver ☐ Other	

Part I: Information about the Child		
1. Child's Information	a) Name:	f) Place of birth:
	b) Date of birth (mm/dd/yyyy):	g) Nationality:

<table>
<tr><td rowspan="3"></td><td>c) Sex: ☐ Male ☐ Female</td><td>h) Ethnicity:</td></tr>
<tr><td>d) Age (yy/mm/dd):</td><td>i) Current school year:</td></tr>
<tr><td>e) Birth order (1st child, 2nd child, etc.):</td><td>j) Number of times the MQ was administered for this child:</td></tr>
<tr><td colspan="2">2. Does the child have any current or past hearing problems?
If yes, please specify:</td><td>☐ Yes ☐ No</td></tr>
<tr><td colspan="2">3. Has hearing ever been tested?
If yes, when?</td><td>☐ Yes ☐ No</td></tr>
<tr><td colspan="2">4. Were there any serious complications during birth?
If yes, please specify:</td><td>☐ Yes ☐ No</td></tr>
<tr><td colspan="2">5. Does the child have any current or past serious health problems?
If yes, please specify:</td><td>☐ Yes ☐ No</td></tr>
<tr><td colspan="2">6. Does the child show any current language problems?
If yes, please specify:</td><td>☐ Yes ☐ No</td></tr>
<tr><td rowspan="4">7. Current Residence</td><td colspan="2">City/Village: State/Province: Country:</td></tr>
<tr><td colspan="2">How long has the child lived at this location?
Years: Months:</td></tr>
<tr><td colspan="2">Who lives with the child at this location?</td></tr>
<tr><td colspan="2">What language(s) is spoken at this location?</td></tr>
</table>

<table>
<tr><td></td><td colspan="4"></td></tr>
<tr><td colspan="5">8. Other Places of Residence (More recent ones first)</td></tr>
<tr><td colspan="3">Location</td><td>How long did the child live there? (years/months)</td><td>What language(s) is spoken there?</td></tr>
<tr><td>City/Town</td><td>State/ Province</td><td>Country</td><td></td><td></td></tr>
<tr><td></td><td></td><td></td><td></td><td></td></tr>
<tr><td></td><td></td><td></td><td></td><td></td></tr>
<tr><td></td><td></td><td></td><td></td><td></td></tr>
<tr><td></td><td></td><td></td><td></td><td></td></tr>
<tr><td colspan="5">9. Does the child visit places where different languages are spoken? ☐ Yes ☐ No
(e.g., visiting relatives in other countries, etc.)
If yes:
• Where?
• How often?
• For how long?
What language(s) is spoken there?</td></tr>
<tr><td colspan="5">10. How shy is the child?
(Please refer to the following scale.)
1 2 3 4 5</td></tr>
</table>

Introverted<br>/Very shy	Extroverted<br>/Outgoing
a) At school/daycare:	
1 2 3 4 5	
b) At home:	
11. Comments:	

Part II: Child Language Information

Section A. Background

1. What languages does the child speak and/or understand (including his/her native language?)

a) Language 1		c) Language 3	
b) Language 2		d) Language 4	

2. When and where did the child first hear each language?

(For language(s) that the child heard from birth, please write “0 (zero)” under age.)

Languages	Language 1	Language 2	Language 3	Language 4
a) Age (year/month)				
b) Place of exposure				

3. When did the child begin to speak each language?

Languages	Language 1	Language 2	Language 3	Language 4
Age (year/month)				

4. How did the child acquire each language?

(e.g., from parents, at school/daycare, through television, from the nanny, etc.)

Language 1	
Language 2	
Language 3	
Language 4	

Section B. Language Exposure

1. How long has the child received or been exposed to (semi-)formal education to learn each language? (e.g., preschool/kindergarten immersion programs, tutoring, etc.) For "Other" please specify the type of education that the child received.

Programs	Preschool (Age) [From , To]	Kindergarten (Age) [From , To]	Tutoring (Age) [From , To]	Other (Age): __________ [From , To]
Language 1				
Language 2				
Language 3				
Language 4				

2. How frequently does the child hear each language in the following situations?

The percentages in each row should add up to 100%. If the child knows two languages and hears both equally, write 50% under both. If the child knows three languages and hears all of them equally, write 33% under all three.[2]

[2]The MQ evaluator should cross-check this information in Supporting Material I – Child Weekly Activities.

<table>
<tr><td colspan="2"></td><td>Language
1 (%)</td><td>Language
2 (%)</td><td>Language
3 (%)</td><td>Language
4 (%)</td></tr>
<tr><td colspan="2">a) At home</td><td></td><td></td><td></td><td></td></tr>
<tr><td colspan="2">b) At school/
day care</td><td></td><td></td><td></td><td></td></tr>
<tr><td rowspan="3">c) At other
places</td><td>1.</td><td></td><td></td><td></td><td></td></tr>
<tr><td>2.</td><td></td><td></td><td></td><td></td></tr>
<tr><td>3.</td><td></td><td></td><td></td><td></td></tr>
<tr><td colspan="2">d) Overall Exposure</td><td></td><td></td><td></td><td></td></tr>
<tr><td colspan="6">e) Comments:</td></tr>
<tr><td colspan="6">3. Do you try to encourage or help the child learn:
☐ all languages
☐ one language over the others
☐ any additional languages
☐ other:
Please, explain:</td></tr>
<tr><td colspan="6">Section C. Comprehension and Production</td></tr>
<tr><td colspan="6">1. Child’s Proficiency Level (Listening/Speaking/Overall)</td></tr>
</table>

Please rate the child's proficiency level using the scales in Appendix II (p. 26).			
	Listening (Oral Comprehension)	Speaking (Oral Production)	Overall Proficiency
Language 1			
Language 2			
Language 3			
Language 4			
e) In your view, is your child's comprehension in each language age appropriate?<br>☐ Yes ☐ Non Comments: ____			
f) In your view, is your child's production in each language age appropriate?<br>☐ Yes ☐ No Comments: ____			

Refer to the scale below when answering Questions 2 and 3 of this section.<br>0 = not at all 2 = so-so 4 = excellent<br>1 = weak 3 = good X = don't know				
**2. Rate the child's ability to <u>understand</u> someone else speaking in these different situations at an age appropriate level.**				
	Language 1	Language 2	Language 3	Language 4

a) Radio				
b) Movies/TV without subtitles or close captions				
c) Spoken instructions				
d) Jokes				
e) Song lyrics				
f) Conversations with friends				
g) Telephone conversations				
h) At school/daycare				
i) At a doctor's office				
**3. Rate the child's ability to speak in these different situations at an age appropriate level.**				
	**Language 1**	**Language 2**	**Language 3**	**Language 4**
a) Conversing with friends				
b) Telling jokes				
c) Counting				

d) Talking on the phone				
e) At school/daycare				
f) At a doctor's office				

Section D. Language Use in Context
**1. How frequently does the child use each language in the following contexts?** The percentages in each row should add up to 100%.If the child knows two languages and uses both equally, write 50% under both. If the child knows three languages and uses all of them equally, write 33% under all three. Also indicate which language the child feels more comfortable using in each situation.

		Language 1	Language 2	Language 3	Language 4	More comfortable using
a) At home						
With Caregiver 1						
With Caregiver 2						
With Additional Caregiver						
With	1.					

siblings	2.					
	3.					
	4.					
With other frequently contacted relatives						
With guests/strangers						
With friends/peers						
**b) At school/daycare**						
With friends/peers						
With teachers						
With guests/strangers						
**c) In the community**						
With friends/peers						
With adults						
**d) At other places**						
•						
•						
e) Entertainment (e.g., TV, movies,						

computer games etc.)					
f) Books					
g) Overall Use					

2. In which language(s) does the child prefer to tell jokes?

Explain/Provide examples if possible.

3. In which language(s) does the child prefer to sing?

Explain/Provide examples if possible.

4. In which language(s) does the child prefer to count?

Explain/Provide examples if possible.

5. Does the child try to avoid situations/places where a specific language is spoken?

☐ Yes ☐ No

Explain/Provide examples if possible.

6. Has there been any change in the child's language use or level of comfort in each language?

☐ Yes ☐ No ☐ Not sure

Explain/Provide examples if possible.

**7. Has the child lost or is the child losing the ability to use any of his/her languages?**<br><br>☐ Yes ☐ No ☐ Not sure<br><br>Explain/Provide examples if possible.

Part III: Family Background Information

1. Caregiver 1's Information	
a) Name:	f) Ethnicity:
b) Sex: ☐ Male ☐ Female	g) Last year of school finished:
c) Age:	h) Current occupation:
d) Place of birth:	i) Previous occupations:
e) Nationality:	j) Relation to the child:

2. Current Residence	City/Village: State/Province:
	Country: Years of stay:

3. Other Places of Residence (More recent ones first)

Country	Years of stay

4. What languages does Caregiver 1 speak and/or understand?

For language(s) that Caregiver 1 learned from birth, please write "0 (zero)" under age.

	Language	Age when Caregiver 1 began learning this	Has Caregiver 1 had any formal education in this

		language	language?
a) Language 1			
b) Language 2			
c) Language 3			
d) Language 4			
**5. Proficiency Level (Listening/Speaking/Overall)**<br><br>Please rate Caregiver 1’s proficiency level using the scales in Appendix II (p. 26).			
	Listening<br><br>(Oral Comprehension)	Speaking<br><br>(Oral Production)	Overall<br><br>Proficiency
a) Language 1			
b) Language 2			
c) Language 3			
d) Language 4			

6. Literacy Level (Reading/Writing :<br><br>N = None; L = Low; M = Middle; H = High)		
	Reading	Writing
a) Language 1	☐ N ☐ L ☐ M ☐ H	☐ N ☐ L ☐ M ☐ H
b) Language 2	☐ N ☐ L ☐ M ☐ H	☐ N ☐ L ☐ M ☐ H
c) Language 3	☐ N ☐ L ☐ M ☐ H	☐ N ☐ L ☐ M ☐ H
d) Language 4	☐ N ☐ L ☐ M ☐ H	☐ N ☐ L ☐ M ☐ H
7. Language(s) spoken to the child:<br><br>If more than one language is spoken, please specify to what extent each one is used.		
8. Language(s) spoken to Caregiver 2:<br><br>If more than one language is spoken, please specify to what extent each one is used.		
9. Language(s) spoken to Additional Caregiver:<br><br>If more than one language is spoken, please specify to what extent each one is used.		
10. Does Caregiver 1 read or tell stories to the child? ☐ Yes ☐ No<br><br>If yes, in which language(s) and how often?<br><br>1.<br><br>2.<br><br>3.<br><br>4.		

11. Caregiver 2's Information	
a) Name:	f) Ethnicity:

b) Sex: ☐ Male ☐ Female	g) Last year of school finished:
c) Age:	h) Current occupation:
d) Place of birth:	i) Previous occupations:
e) Nationality:	j) Relation to the child:

<table>
<tr><td rowspan="2">12. Current Residence</td><td>City/Village: State/Province:</td></tr>
<tr><td>Country: Years of stay:</td></tr>
</table>

<table>
<tr><td colspan="2">13. Other Places of Residence (More recent ones first)</td></tr>
<tr><td>Country</td><td>Years of stay</td></tr>
<tr><td></td><td></td></tr>
<tr><td></td><td></td></tr>
<tr><td></td><td></td></tr>
<tr><td></td><td></td></tr>
</table>

<table>
<tr><td colspan="4">14. What languages does Caregiver 2 speak and/or understand?
For language(s) that Caregiver 2 learned from birth, please write “0 (zero)” under age.</td></tr>
<tr><td></td><td>Language</td><td>Age when Caregiver 2 began learning this language</td><td>Has Caregiver 2 had any formal education in this language?</td></tr>
<tr><td>a) Language 1</td><td></td><td></td><td></td></tr>
<tr><td>b) Language 2</td><td></td><td></td><td></td></tr>
</table>

c) Language 3			
d) Language 4			

15. Proficiency Level (Listening/Speaking/Overall)

Please rate Caregiver 2's proficiency level using the scales in Appendix II (p. 26).

	Listening (Oral Comprehension)	Speaking (Oral Production)	Overall Proficiency
a) Language 1			
b) Language 2			
c) Language 3			
d) Language 4			

16. Please rate Caregiver 2's ability to speak and understand each language using the following scale.

0 = not at all 1 = weak 2 = so-so 3 = good 4 = excellent X = don't know

	Speak	Understand
a) Language 1		
b) Language 2		
c) Language 3		
d) Language 4		

17. Literacy Level (Reading/Writing :

N = None; L = Low; M = Middle; H = High)

	Reading	Writing
a) Language 1	☐ N ☐ L ☐ M ☐ H	☐ N ☐ L ☐ M ☐ H
b) Language 2	☐ N ☐ L ☐ M ☐ H	☐ N ☐ L ☐ M ☐ H
c) Language 3	☐ N ☐ L ☐ M ☐ H	☐ N ☐ L ☐ M ☐ H
d) Language 4	☐ N ☐ L ☐ M ☐ H	☐ N ☐ L ☐ M ☐ H

18. Language(s) spoken to the child:

If more than one language is spoken, please specify to what extent each one is used.

19. Language(s) spoken to Caregiver 1:

If more than one language is spoken, please specify to what extent each one is used.

20. Language(s) spoken to Additional Caregiver:

If more than one language is spoken, please specify to what extent each one is used.

21. Does Caregiver 2 read or tell stories to the child? ☐ Yes ☐ No

If yes, in which language(s) and how often?

1.

2.

3.

4.

22. Additional Caregiver's Information	
a) Name:	f) Ethnicity:
b) Sex: ☐ Male ☐ Female	g) Last year of school finished:

c) Age:	h) Current occupation:
d) Place of birth:	i) Previous occupations:
e) Nationality:	j) Relation to the child:

**23. Current Residence**	City/Village:	State/Province:
	Country:	Years of stay:

24. Other Places of Residence (More recent ones first)

Country	Years of stay

25. What languages does Additional Caregiver speak and/or understand?

For language(s) that Additional Caregiver learned from birth, please write "0 (zero)" under age.

	Language	Age when Additional Caregiver began learning this language	Has Additional Caregiver had any formal education in this language?
a) Language 1			
b) Language 2			

c) Language 3			
d) Language 4			

26. Proficiency Level (Listening/Speaking/Overall)

Please rate Additional Caregiver's proficiency level using the scales in Appendix II.

	Listening (Oral Comprehension)	Speaking (Oral Production)	Overall Proficiency
a) Language 1			
b) Language 2			
c) Language 3			
d) Language 4			

27. Please rate Additional Caregiver's ability to speak and understand each language using the following scale.

0 = not at all 1 = weak 2 = so-so 3 = good 4 = excellent X = don't know

	Speak	Understand
a) Language 1		
b) Language 2		
c) Language 3		
d) Language 4		

28. Literacy Level (Reading/Writing :

N = None; L = Low; M = Middle; H = High)

	Reading	Writing
a) Language 1	☐ N ☐ L ☐ M ☐ H	☐ N ☐ L ☐ M ☐ H
b) Language 2	☐ N ☐ L ☐ M ☐ H	☐ N ☐ L ☐ M ☐ H
c) Language 3	☐ N ☐ L ☐ M ☐ H	☐ N ☐ L ☐ M ☐ H
d) Language 4	☐ N ☐ L ☐ M ☐ H	☐ N ☐ L ☐ M ☐ H

29. Language(s) spoken to the child:

If more than one language is spoken, please specify to what extent each one is used.

30. Language(s) spoken to Caregiver 1:

If more than one language is spoken, please specify to what extent each one is used.

31. Language(s) spoken to Caregiver 2:

If more than one language is spoken, please specify to what extent each one is used.

32. Does Additional Caregiver read or tell stories to the child? ☐ Yes ☐ No

If yes, in which language(s) and how often?

1.

2.

3.

4.

33. Siblings' Information

For 34(f), please refer to the overall proficiency scale in Appendix II (p. 26).

For 34(g) – 34(k), if more than one language is spoken, please specify to what extent each one is used.				
	**Sibling 1**	**Sibling 2**	**Sibling 3**	**Sibling 4**
a) Name				
b) Sex				
c) Age				
d) Birth order: (1st child, etc.)				
e) Language(s) spoken and/or understood				
f) Overall Proficiency (Scale of 0–5)				
g) Language(s) spoken to the Caregiver 1				
h) Language(s) spoken to the Caregiver 2				

i) Language(s) spoken to Additional Caregiver				
j) Language(s) spoken to the child				
k) Language(s) spoken to other siblings				

34. Other Frequently Contacted People

(e.g., relatives, friends, neighbors, nannies, etc.)

If more than one language is spoken, please specify to what extent each one is used.

Relation to the child	Language(s) spoken to the child
1.	
2.	
3.	
4.	
5.	
6.	

7.	
**35. In what language(s) is written material available at home?**<br><br>(e.g., books, newspapers, periodicals)<br><br>1.<br><br>2.<br><br>3.<br><br>4.	
**36. Has there been any change in the languages spoken in the family?**<br><br>Explain/Provide examples.	
**37. Comments:**	

Part IV: Code-Switching (Language Mixing)

- **Language mixing**: Changing from one language to the other during speech.
- **Types of language mixing**:
  - **Word-switch**: switching **one word** in a sentence from one language to the other. (e.g., If you go to the store, can you bring back some ***huevos (eggs)***?)
  - **Sentence/Phrase switch**: switching **a group of words** in a sentence from one language to the other. (e.g., I'm going to watch TV ***en el cuarto de mi mamá. (in my mom's room)***.)

1. Does the child change from one language to the other when he/she speaks with the following people?

	Yes	No	Word switch	Phrase switch
a) Caregiver 1	☐	☐	☐	☐
Languages Mixed:				
b) Caregiver 2	☐	☐	☐	☐
Languages Mixed:				
c) Additional Caregiver	☐	☐	☐	☐
Languages Mixed:				
d) Siblings	☐	☐	☐	☐
Languages Mixed:				
e) Relatives	☐	☐	☐	☐

Languages Mixed:				
f) Friends/peers	☐	☐	☐	☐
Languages Mixed:				
g) Community Members	☐	☐	☐	☐
Languages Mixed:				
h) Overall	☐	☐	☐	☐
Languages Mixed:				
i) If you can, please give some examples of the child's language mixing.				

2. Do the following people change from one language to the other when they speak to the child?

	Yes	No	Word switch	Phrase switch
a) Caregiver 1	☐	☐	☐	☐
Languages Mixed:				
b) Caregiver 2	☐	☐	☐	☐

Languages Mixed:				
c) Additional Caregiver	☐	☐	☐	☐
Languages Mixed:				
d) Siblings	☐	☐	☐	☐
Languages Mixed:				
e) Relatives	☐	☐	☐	☐
Languages Mixed:				
f) Friends/peers	☐	☐	☐	☐
Languages Mixed:				
g) Community Members	☐	☐	☐	☐
Languages Mixed:				

Part V: Reading/Writing (not applicable to very young children)

Reading

1. Does the child know how to read? ☐ Yes ☐ No

2. If yes, how well does the child read for his/her age?

	Perfectly	Well	Sufficiently	Almost nothing
a) Language 1				
b) Language 2				
c) Language 3				
d) Language 4				

e) Comments:

Writing

3. Does the child know how to write? ☐ Yes ☐ No

4. If yes, how well does the child write for his/her age?

	Perfectly	Well	Sufficiently	Almost nothing
a) Language 1				
b) Language 2				
c) Language 3				
d) Language 4				

e) Comments:

Refer to the scale below when answering Questions 5 and 6 of this section.<br>0 = not at all 1 = weak 2 = so-so 3 = good 4 = excellent X = don't know				
**5. Rate the child's ability to <u>write</u> the following at an age appropriate level.**				
	Language 1	Language 2	Language 3	Language 4
a) Informal correspondence (letter/e-mail to friends/family)				
b) Homework/ composition				

**6. Rate the child's <u>understanding</u> of these written materials at an age-appropriate level.**				
	Language 1	Language 2	Language 3	Language 4
a) Magazines/internet				
b) Books/textbooks				
c) Letters/e-mail				

Part VI: Comments by Interviewer/Questions or Notes From Parents/Caregivers

Supporting Material I – Child Weekly Activities

Please indicate how the child spends a typical week on an hourly basis. Include where the child spends his/her time, with whom, and what language(s) is spoken in that environment.

	Monday	Tuesday	Wednesday	Thursday	Friday	Saturday	Sunday
6:00AM– 8:00AM							
8:00AM– 10:00AM							
10:00AM– 12:00PM							
12:00PM– 2:00PM							
2:00PM– 4:00PM							
4:00PM– 6:00PM							
6:00PM– 8:00PM							
8:00PM– 10:00PM							

Comments:

Supporting Material II –

Proficiency Scale for Oral Comprehension (Listening)

0 = No understanding

1 = Understands some single words only – no more than 10 words approximately

2 = Understands some single words only – more than 10 words

3 = Understands some sentence combinations, usually with repetition or other assistance

4 = Good understanding of conversations and other language domains (e.g., TV, movies, story reading)

5 = Native-like Proficiency

6 = Unknown

Proficiency Scale for Oral Production (Speaking)

0 = No knowledge of the language

1 = Knowledge of single words mainly – no more than 10 words approximately

2 = Knowledge of single words mainly – more than 10 words

3 = Combines words to create simple sentences containing only a few words (e.g., two-word utterances); Speaks with pauses or phonological errors and is sometimes not understood

4 = Combines words to create longer sentences; Speaks sufficiently and is almost always understood

5 = Native-like proficiency

6 = Unknown

Overall Proficiency Scale

0 = Cannot speak and cannot understand the given language

1 = Cannot speak but sometimes understands the general idea of what is being said

2 = Limited proficiency and limited vocabulary

3 = Good proficiency

4 = Native-like proficiency

5 = Unknown

Supporting Material III –

Researcher's Judgment on Child's Language Proficiency

Which type of questionnaire was administered for this child?

☐ Long Form

☐ Short Form

<table>
<tr><td colspan="4">Upon filling out the VLL Child MQ Evaluation Form, the researcher should answer the following section based on his/her own assessment.</td></tr>
<tr><td colspan="4">Child's Proficiency Level (Listening/Speaking/Overall)
Please rate the child's proficiency level using the scales in Appendix II (p. 26).
(L1: 1st acquired language, L2: 2nd language, L3: 3rd language, L4: 4th language)</td></tr>
<tr><td></td><td>Listening
(Oral Comprehension)</td><td>Speaking
(Oral Production)</td><td>Overall
Proficiency</td></tr>
<tr><td>a) L1:</td><td></td><td></td><td></td></tr>
<tr><td>b) L2:</td><td></td><td></td><td></td></tr>
<tr><td>c) L3:</td><td></td><td></td><td></td></tr>
<tr><td>d) L4:</td><td></td><td></td><td></td></tr>
<tr><td colspan="4">e) In your view, is the child's comprehension in each language age appropriate?
☐ Yes ☐ No Comments:</td></tr>
<tr><td colspan="4">f) In your view, is the child's production in each language age appropriate?
☐ Yes ☐ No Comments:</td></tr>
</table>
