# Supplementary material for: Acquisition of a new language: an enriched case study documents language growth without external input in a young Korean child’s acquisition of English
Source: Front Hum Neurosci. 2024 Nov 27;18:1456054. doi: 10.3389/fnhum.2024.1456054 (PMC11631620; doi:10.3389/fnhum.2024.1456054)
Supplement: Supplementary file 2 [file Data_Sheet_2.DOC]

**General Observation Procedures**

1.Students/researchers are trained in behavioral observation methods

2.Students/researchers are trained in metadata recording and organization

3.Each child observed is entered into a master database with recordings based on each observation

4.Students/researchers are taught ‘general observation’ format and principles of analysis

5.Full Transcripts are created for each session and reliability checked by a second researcher.

6.Full Transcripts are analyzed (each utterance is coded) according to leading criteria (pragmatic of speech based) and reliability checked by a second observer.

7.Distilled transcripts involving only speech data are created and analyzed for criteria of interest.

**Examples**

**Multilingualism Case Studies Procedures**

**A.General Online Protocol**

**(1)General Online Protocol**

**Observer: __________**

**Date: ___________**

| Subject | Time | General Behavior | Speech Production | Comprehension |
| --- | --- | --- | --- | --- |
|  |  |  |  |  |
|  |  |  |  |
|  |  |  |  |

**(2) Example**

**Observer: _____**Soon**______**

**Date: _____**

| Subject | Time | General Behavior | Speech Production | Comprehension |
| --- | --- | --- | --- | --- |
| MJ | 09:40am ~ 09:45am | Nathan pushed him so he cried, Sit still next to a teacher |  | “do you love mommy this much?”, “do you want to go in the ship with Julius?”  (🡪 shook his head for both Qs) |
|  | 09:45am ~ 09:50am | Play with peers imitating cats |  | “do you want to be a baby/boy?”, “do you want to play with Bailey?”  (🡪 shook his head again) |
|  | 09:50am ~ 09:55am | Crawl imitating a cat | “no dangerous” |  |
|  | 09:55am ~ 10:00am | Cry again |  |  |
|  |  |  |  |  |

**B. Example Session Transcription (Full)**

Case ID of Child: MJ

Sex: Male

Date of Birth:

Age During Interview: 3 years, 4 months, 7 days

Session #: 5 (General Observation)

Date of Session:

Length of Session: 17 minutes, 51 seconds

Location of Session: ECC

Transcriber:

Reliability Checked with X

Setting: Students are seated in a circle, each with an alphabet block depicting one letter (and a picture of a word corresponding to that letter)

(2:06) **Students with block “J”**

Teacher A: Carol, would you like to show us your letter?

Teacher B: J

Boy A: Little J

Teacher A: Lowercase J. Could (XX)?

MJ: What’s lower?

(2:20) **Student with block “T”**

Teacher A: Julius, would you like to show us your letter?

Boy B: My letter is letter G. L^

Child X: T.

Teacher A: T?

Boy B: This (XX). And (XX)

Teacher A: it has a t^?

MJ: J>

Teacher: A top?

MJ: J> J f-> J for> J for me, right? (Turns to teacher, puts hand on leg).

(4:01) **MJ’s letter B**

Teacher A: MJ can you show us your letter?

Minghi: (Looks at block and shows it to Teacher A)

Teacher A: What letter is that (positions block in MJ’s hand so other students can see it)?

Teacher A (to Boy C): Remember how sad Doris was?

Teacher B: It’s MJ’s turn now. {You need to be listening to MJ}

Teacher A: {whispers to MJ.}

MJ: (mouths “B” repeatedly)

Teacher A: A B

(5:56) **Teacher reads story featuring each of the letters**

Teacher A: B (turns to MJ and guides his arm toward the center area), “B” tumbled into the bath.

Student X : {MJ, put it back!}

Teacher A: {MJ, can you put B in there?}

MJ: (crawls toward center to return block B) X!

(6:40) **MJ blocks the view of the book**

MJ: (puts his hand on the teacher and sits up)

Teacher A: MJ!

(7:35) **Teacher on page “P”**

MJ: (tries to stand up) No, I c(X) s(X).

Teacher A: Do you want to move, MJ? Do you want to move over by Mira (points to left)?

MJ: (gets up and moves in that direction)

(7:58) **Teacher on page “R”**

Teacher A: R rolled away^

MJ: I can’t see (XX).

Teacher A: on a roller skate.

(8:10) Teacher on page S)

Teacher A: {S went to play in the sandbox.}

MJ: {XXX} (Tries to push Girl A to the side).

(9:34) **Teacher begins post-story discussion**

Teacher A: And MJ, where do you think the letters might go next?

MJ: (Pauses) Uuh, cookies.

Teacher A: In the cookies?

MJ: Yea.

12:06

(Minghi reaches for the basket, picks up a block, and shows to teacher)

(12:52) **Students sing an alphabet song (interacting with self- do not count)**

Teacher & children sing Alphabet song

MJ: No response

Children sing alphabet song w/o teacher

MJ: Makes funny faces (no response)

Teacher and children sing Twinkle

MJ: Lies on his back (no response)

15:24

(MJ nudges girl with feet)

(16:16) **Nonverbal Initiation**

MJ: (Lunges forward and ends up in front of girl.)

Girl B: (Lunges back)

(16:37) **MJ tries to walk to the other room**

Teacher A: MJ?

MJ: I have to pee.

Teacher A: Huh?

MJ: I (X) to (X)

Teacher X: You have to go? (XXX) Gotta go? OK

(17:17) **MJ returns from bathroom/students interact informally**

MJ: (faces camera): (XXX)

17:30 **Girl A is pushing MJ**

MJ: (XXX) (pushes back)

XX=uninterpretable

**C. Example Session Transcription - Language**

Case ID of Child: MJ

Sex: Male

Date of Birth:

Age During Interview: 3 years, 4 months, 7 days

Session #: 5 (General Observation)

Date of Session:

Length of Session: 17 minutes, 51 seconds

Location of Session: ECC

Transcriber:

Reliability Checked

**Subject Only English Verbal Utterances**

*If nothing comes before or after a parenthetical phrase, that indicates no verbal response took place.

**What’s lower?**

- 3 syllables, 4 morphemes, 3 words

**J> J> J f-> J for> J for me, right?**

- 4 syllables, 4 morphemes, 4 words

**X!**

- 1 syllable, 1 morpheme, 1 word

**No, I c(X) s(X).**

- Yes/No category- Do not count
- Unclear/Fragment- Do not count

**I can’t see (XX).**

- Unclear/Fragment- Do not count

**{XXX}**

- Unclear/Fragment- Do not count

**Uuh, cookies.**

- Filler- do not count
- 2 syllables, 2 morphemes, 1 word

**Yea.**

- Yes/no category- Do not count

**I have to pee.**

- 4 syllables, 4 morphemes, 4 words

**I (X) to (X)**

- Unclear/Fragment – Do not count

**XXX**

- Unclear/Fragment – Do not count

**(XXX)**

- Unclear/Fragment – Do not count

**D. General Principles for Data Analysis (Yarden Kedar)**

| **I. Practice with Language** |
| --- |
| 1. If the child’s peers are having a conversation and the child was within earshot, how and when does the child respond? |
| 2. If the child doesn’t understand something that is said to him/her, how does the child react? |
| 3. When the child plays alone, does the child speak to himself/herself? Does the child speak as if s/he were talking to somebody as in a play? |

| **e** |
| --- |
| 1. If the child tries to tell something and the listener doesn’t understand what the child says, what does the child do? |
| 2. When the child’s peers are having a conversation, does the child ever try to join the conversation? If so, how does the child try to do so? |
| 3. When the child tries to speak to one specific person (not in a conversation group), how does the child approach? |
| **II. Trial with Language** |
| 1. If the child tries to tell something and the listener doesn’t understand what the child says, what does the child do? |
| 2. When the child’s peers are having a conversation, does the child ever try to join the conversation? If so, how does the child try to do so? |
| 3. When the child tries to speak to one specific person (not in a conversation group), how does the child approach? |

| **III. Context Dependency**  1. Are there any specific peers or people to whom the child seems to feel more comfortable to speak? |
| --- |
| 2. Do any social conditions affect the way the child speaks (e.g., even though the child is likely to speak to a certain peer, s/he wouldn’t speak to that peer if the peer is with others)? |
| 3. Are there any topics the child is more likely to talk about or any activities, in which the child is more likely to speak? |
| 4. If the trial to join a conversation or to make sense to someone was failed, how does the child seem to feel? |
| 5. When it gets to the point of expressing her/his emotion in general, how does the child display it? |
| 6. If the child becomes quite emotional (e.g., upset or lugubrious) and has to speak at the same time, how does the child explain the situation or surroundings? |
